# Cardiotoxicity of Cadmium and Its Effects on Heart Efficiency During Early and Late Chick Embryogenesis

**DOI:** 10.1007/s12012-024-09894-x

**Published:** 2024-07-24

**Authors:** Reda A. Ali, Eatemad A. Awadalla, Amal S. Hamed, Dalia Elzahraa F. Mostafa

**Affiliations:** 1 Zoology Department, Faculty of Science, Assiut University, Assiut, 71516 Egypt; 2 Zoology Department, Faculty of Science, Aswan University, Aswan, 81528 Egypt

**Keywords:** Cadmium, ECG, Respiratory rate, Cardiomyocytes, Endomysia, Edema

## Abstract

Cadmium (Cd) is a dangerous heavy metal that is non-degradable in the environment. Many organs can accumulate Cd and adversely affect organ function and health. Cd is considered as a teratogenic and embryotoxic agent. This study aims to evaluate the teratogenicity of Cd at concentrations lesser than the permissible and its effects on the heart during chick embryogenesis. Fertilized eggs of the chick *Gallus domesticus* were divided into; control, saline injected and four experimental groups injected with single doses of 5, 25, 50 or 75 µM of CdCl_2_. Histological observations of the heart before hatching and the cardiomyocytes after hatching were recorded. Morphometric measurements of heart chambers were achieved at 3, 4 and 6 days of incubation. Electrocardiograph and respiratory rate were recorded at tenth day. Different cardiac problems had been brought on by Cd. In comparison to controls, the heart looked much larger, and in certain cases, growth retardation was seen. Degeneration in heart walls and malformations of dorsal aorta were noticed. Morphometrically, the width and wall thickness of heart chambers showed significant changes. Heart beats and respiratory rate significantly decreased compared to control. The cardiotoxic effect of Cd on heart compartments structure and function was dose dependent. One of Cd toxicity is its ability to induce cellular oxidative stress. The heart in particular is sensitive to oxidative stress. Cardiac oxidative stress might intensify heart failure and promote disease progression. Calcium is one of the components that is needed for normal heart work. Cd might interfere with calcium metabolism by removing it from the body.

## Introduction

One major heavy metal that is not necessary and is found in all parts of the environment, including the soil, water, air and food, is cadmium (Cd). About ten times less Cd is added to the environment by natural sources compared to anthropogenic sources [1]. Food is the major source of human exposure to Cd, as plants can absorb Cd from the soil. Subsequently, Cd can easily incorporate into different plant originated foodstuff [2].

Cd can produce major environmental hazards such as embryotoxicity, carcinogenicity and teratogenicity in developing embryos, which can lead to serious abnormalities such as body weight loss, micromelia, microphthalmia and haemorrhages [3]. A recent study revealed that Cd was the most toxic pollutant that affected embryonic development and behavior of *Planorbella duryi* embryos at concentrations ranging from 0 to 10 mg/L, suggesting that exposure to Cd toxicity may cause extended effects that might touch the structure of population or community [4]. Cd plays its role as a teratogen by causing structural and functional changes in the placenta [5]. 75% of the embryos of Japanese medaka (*Oryzias latipes*), exposed to 10 mg/L Cd group were not able to inflate their swim bladders [6].

Because of the low rate of excretion of Cd, an excessive amount of it accumulates over time in various organs, causing a variety of deleterious consequences on animal health, including hepatotoxicity [7], nephrotoxicity [8], neurotoxicity [9] and especially cardiotoxicity [10].

Delivering nutrients, supplying oxygen, and discarding waste products are all performed via the cardiovascular system [11]. Many research studies have demonstrated that several environmental pollutants can cause disorders of the cardiovascular system [12]. Increasing number of data indicated that there is a close connection between Cd-exposure and cardiovascular diseases, like congenital heart disease [13], hypertension [14] even cancer [15]. One of the first organs in the growing embryo that records activity is the heart. It is clear that research into the effects of Cd on the developing embryo heart is crucial, especially since Cd is cardiotoxic in small doses that have no negative effect on other organs [16].

The average Cd intake in food generally varies between 8 and 25 µg/day [17], while in the European countries, it was estimated as 10–30 µg/day [18]. The tolerable daily intake was estimated by FAO as 1 µg/kg of body weight [19]. Maximum contaminant permitted level for Cd in drinking water is 0.005 mg/L [20]. LC_50_ was estimated to be 6 mg/L for the marine medaka, *Oryzias melastigma* [21]. The toxicity of various influences on developing embryos can be studied using chicken embryos as a model. Chick embryos are a common subject of such investigations due to their development outside the mother’s body [22–26]. The LD_50_ of Cd in chick embryo was found to be 3.9 µg Cd/egg [25]. Therefore, the objective of the current study was to evaluate the effects of *in ovo* injection of Cd in concentrations greatly lower than the permissible levels in drinking water on the heart structure, heart work and respiratory rate of chick embryo during its development.

## Materials and Methods

### Dose Preparation

The dose was prepared by dissolving cadmium chloride (CdCl_2_) (LOBA CHEMIE PVT. LTD, INDIA) in saline solution (NaCl) 8.5%. By diluting with the solvent, different concentrations (5, 25, 50 and 75 µM) of CdCl_2_ were prepared.

### Chick Embryos

Fertilized eggs of the *Gallus domesticus* (Dandrawi strain) chicks were acquired from Faculty of Agriculture farm, Assiut University. An electrical incubator with temperature control was used to artificially incubate all of the embryological materials required for the research. The incubator was located in a well-ventilated place and was accurately adjusted at 37.5 ± 0.1 °C before use. Both the trays of the eggs and inside of the incubator were thoroughly cleaned using dettol and 70% ethyl alcohol. Sterilization was carried out using Biocidal ZF™ WAK-Chemie Medical GmbH reagents. Labeled fertile eggs were positioned vertically in the incubator’s trays. Ventilation was allowed in the incubating chamber. Relative humidity was automatically adjusted at 52%. Until the designated operating time, incubated eggs were automatically rotated from side to side roughly every two hours. The incubator used in the present study belongs to PTO, Egypt, model C5, in Experimental Embryology Lab, Department of Zoology, Faculty of Science, Assiut University.

### Eggs Preparation & Fixation

Eggs were thoroughly cleaned with 70% ethyl alcohol. A hole was done at the blunt area of the egg. Injection was carried out by means of a micropipette. Prior to incubation, injections were carried out. After that, sealing tape was used to close the opening. The eggs were incubated and then they were taken out at intervals.

The incubated eggs were carefully opened under physiological saline solution. Embryos were delicately separated from the yolk and membranes, transported to a fresh saline solution for washing before being fixed in formal alcohol (60 ml absolute ethyl alcohol, 30 ml neutral formalin 10% and 10 ml glacial acetic acid) for histological studies. After fixation, the specimens were preserved in 70% ethyl alcohol.

### Experimental Design

The 210 eggs weighing 50 ± 2 gm/egg were randomly and equally divided into six groups (*n* = 35/group):

- The control group was left untreated.
- The positive control group received 50 µl of saline solution as a solvent.
- Four experimental groups: received one injection per egg; each one was 50 µl of different doses of CdCl_2_ (5, 25, 50 and 75 µM) respectively, in saline solution.

These doses was chosen based on a previous study [27].

### Histological Studies

Embryos at 33, 48, 72, 96 and 144 h (*n* = 90 “3 embryos/age/group”) of incubation (stages 10, 13, 19, 23 and 29 respectively) according to Hamburger& Hamilton scale [28] and hearts of twenty one days (after hatching-stage 46) (*n* = 3 hearts/group) were harvested. The choice of such embryonic ages based on the appearance of distinct characteristics features of heart development, where heart clearly starts beating at 33 h. Heart tube twisting is obviously seen at 48 h. Fusion of dorsal aortae is a characteristic feature of 72 h. Thickening of epimyocardium and lose of tubular nature of heart characterize the age of 96 h. Differentiation of epimyocardium into epicardium and myocardium characterizes the age of 144 h.

Following harvesting, the embryos were fixed, dehydrated using ascending concentrations of ethyl alcohol (70, 90 and 100%), cleared in cedar wood oil, chloroform-washed, embedded in paraffin wax and 7 micron-thick transverse serial sections were prepared for the early stages, while 5 micron-thick transverse serial sections were prepared for the heart at the age of 21 days. The staining procedures of Harris haematoxylin and eosin were applied according to Drury & Wallington [29]. An average number (15–20) of histological sections for each stage were microscopically examined.

Histopathological examination was performed under a high-power light microscope (Olympus BX43F, Tokyo 163–0914, Japan). Image analysis was performed using a personal computer, a camera, software (Olympus DP74 Tokyo, 163–0914, Japan) at the Zoology Department, Faculty of Science, Aswan University.

### Heart Chambers and Dorsal Aorta Measurements

After (72, 96, 144 h) of incubation, the various heart components were identified by histological examinations. Morphometric measurements were carried out of the lumen and wall for each of the truncus arteriosus, ventricle, atrium, sinus venosus and dorsal aorta. An average number (22–36) sections were examined for morphometric measurements. Digital images were obtained under 20× magnification for (72 h) and 10× magnification for (96 and 144 h) heart sections, using a digital camera connected to Olympus light microscope. Each compartment of the heart in each group was measured in a minimum 10–12 replicates. Morphometric analysis was performed using the image analysis software “Entry CellSens”.

### Heart Rate Measurement

At the tenth day of incubation (stage 36), heart beats and respiratory rates were recorded using electrocardiograph (Mindray MEC 1200) as shown in the sketch. Electrode pads were adjusted and optimized at the egg shell at five points to get the most obvious and clear pulsation waves. Heart beats and respiratory rate were measured for 20 embryo/group.

**Figure:**
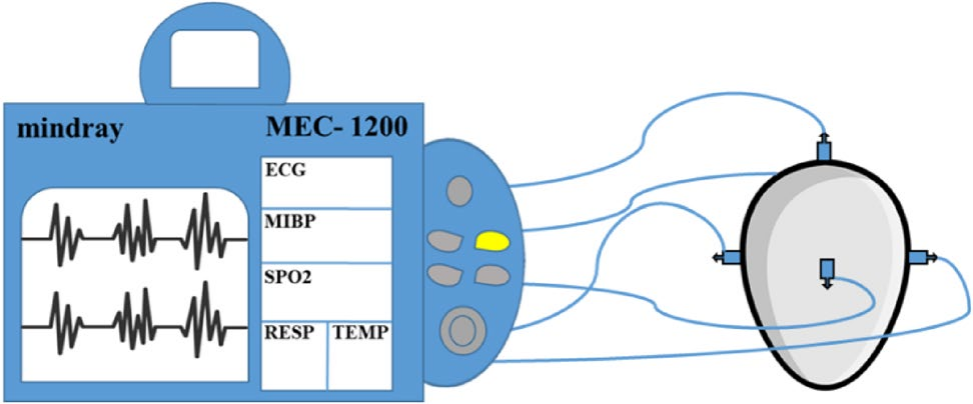
Sketch (1): Electrocardiographing of chick embryo inside the egg shell

### Statistical Analysis

The data were reported as mean ± SE. Column statistics and one-way analysis of variance were used to statistically examine the data and the Newman-Keuls multiple comparison test were used as a posttest. These analyses were carried out using Prism software for windows, version 6.0 (Graph pad software Inc., San Diego, California, USA) and Excel (Microsoft office 10). Differences were considered to be significant when *P* < 0.05.

## Results

### Histological Investigation

#### At the Embryonic Age of 33 h

**Fig. 1:**
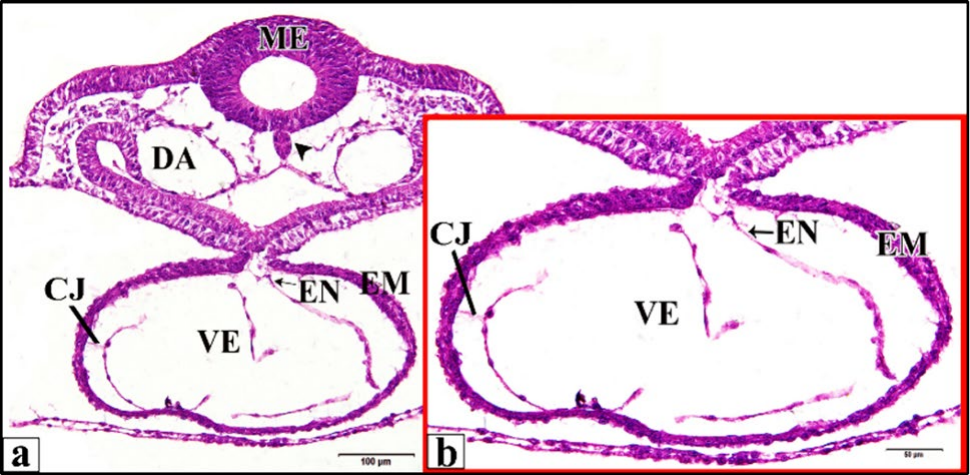
**a** A photomicrograph of a transverse section of a normal heart of 33 hours chick embryo showing its chamber and membranes. **b** A magnification of the ventricle (VE). Mesencephalon (ME), notochord (arrow head), dorsal aorta (DA), epimyocardium (EM), (CJ) cardiac jelly, endocardium (EN). H & E stain

As the control or saline-injected embryo develops, during the age from 24 h to 33 h, the paired heart primordia are brought ventrally together in the mid-line and combine to produce a single straight, double-walled tube. Where the anterior intestine portal’s lateral edges have been undergoing concrescence, stretching the foregut caudally and involving the heart area. The left and right endocardial primordia tubes (a single cell in thickness) have begun to move in the direction of each other underneath the recently constructed fore-gut floor. Each of the original mesodermic thickenings (that appeared in earlier ages) becomes applied to the lateral aspects of the endocardial tubes as the epimyocardial primordium (which is destined to give rise to the external coat of the heart “epicardium” and to the heavy muscular layers of the heart “myocardium”). At the same time the epimyocardial areas of the mesoderm are brought together first ventrally and then dorsally to the endocardium, where the splanchnic mesoderm of the opposite sides of the body comes together dorsal and ventral to the heart. It forms double layered supporting membranes called respectively the dorsal mesocardium and the ventral mesocardium. The ventral mesocardium is a transient anatomical feature that vanishes virtually immediately upon formation. Throughout the remaining several hours of incubation, the majority of the dorsal mesocardium vanishes. Outside the endocardial primordium, between it and the epimyocardial there was a relatively thick layer of cardiac jelly (Fig. 1).

**Fig. 2:**
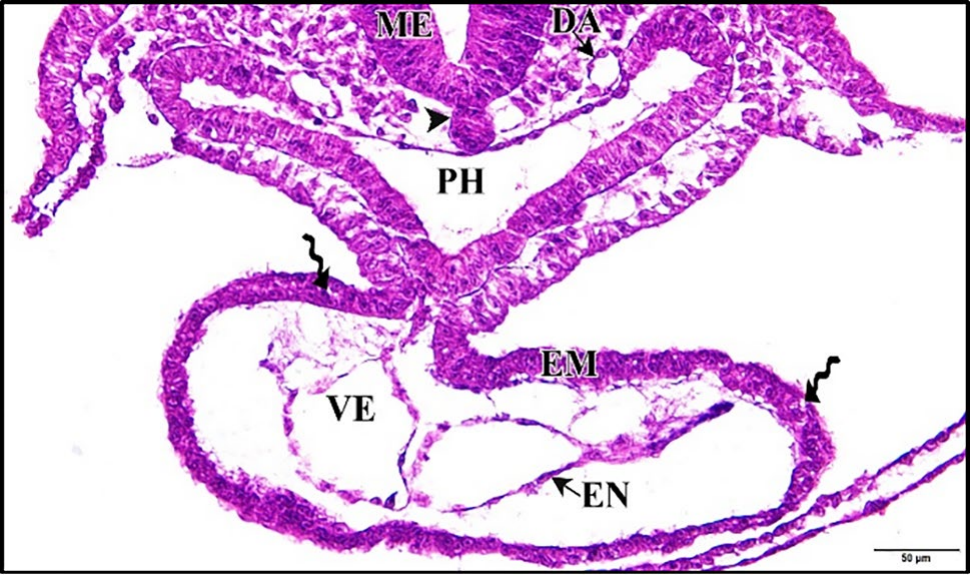
A photomicrograph of a transverse section of a chick embryo treated with 5 µM CdCl_2_ after 33 hours of incubation showing small heart with thick epimyocardium (EM), the spaces between the mesenchymal cells of the epimyocardium (wavy arrows) and two small dorsal aorta (DA). Mesencephalon (ME), notochord (arrow head), pharynx (PH), ventricle (VE), endocardium (EN). H & E stain

**Fig. 3:**
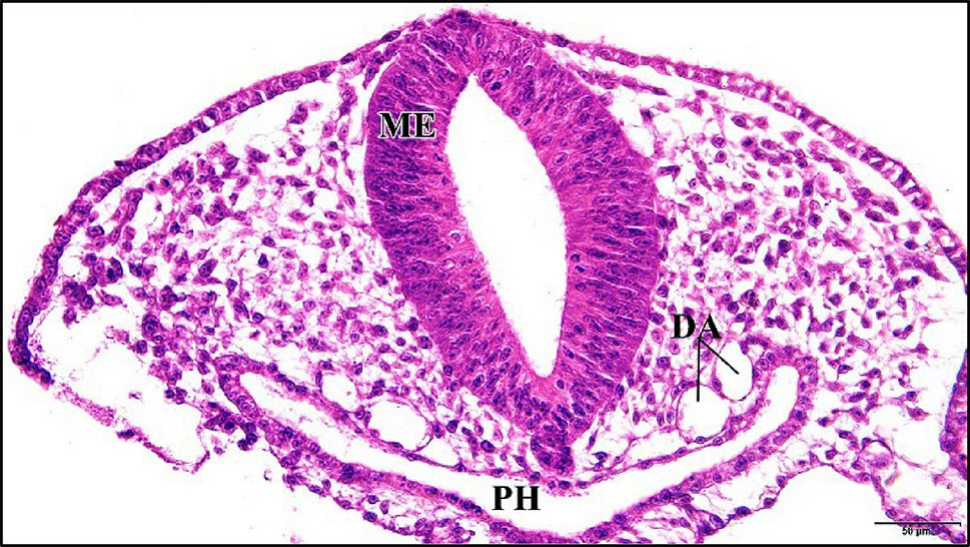
A photomicrograph of a transverse section of a chick embryo treated with 5 µM CdCl_2_ after 33 hours of incubation showing the division of dorsal aorta (DA) into two parts. Mesencephalon (ME), pharynx (PH). H & E stain

The 5 µM CdCl_2_ treated heart was small in size and its epimyocardium was thicker than control. This thickening is due to appearance of spaces in between the mesenchymal cells of the epimyocardium rather than addition of new cells (Fig. 2). The two dorsal aortae were smaller in size and in some regions of the embryo they divide into two parts (Figs. 2 and 3).

25 µM CdCl_2_ treated embryos showed growth retardation. So, the heart was not formed yet.

**Fig. 4:**
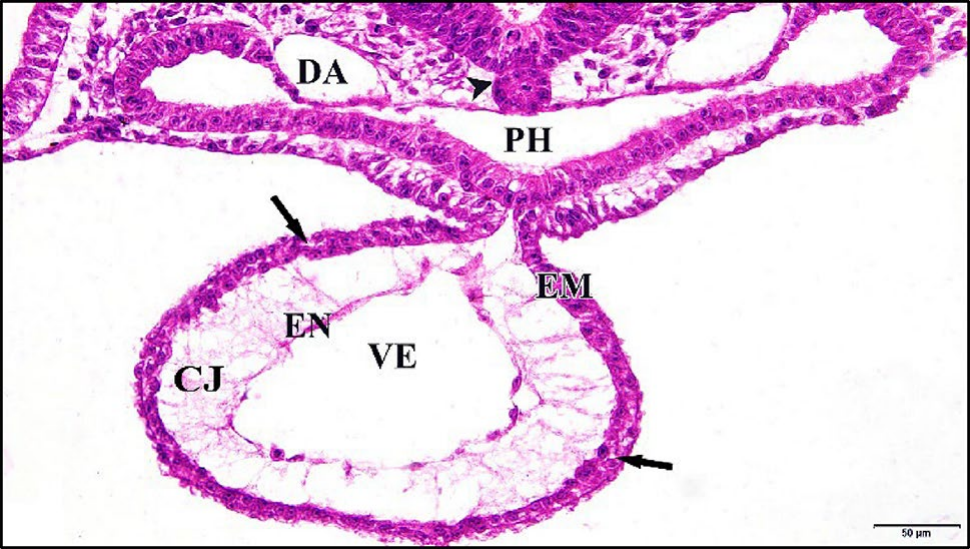
A photomicrograph of a transverse section of a chick embryo treated with 50 µM CdCl_2_ after 33 hours of incubation showing small heart and its thick epimyocardium (EM) and the wide space between the mesenchymal cells of the epimyocardium (arrows). Dorsal aorta (DA), notochord (arrow head), pharynx (PH), ventricle (VE), (CJ) cardiac jelly, endocardium (EN). H & E stain

50 µM CdCl_2_ treated heart was small in size and its epimyocardium wall was thick. Appearance of wide spaces in between the mesenchymal cells of the epimyocardium was obviously noticed rather than addition of new cells. The cardiac jelly in between the endocardium and the epimyocardium was obviously noticed (Fig. 4).

**Fig. 5:**
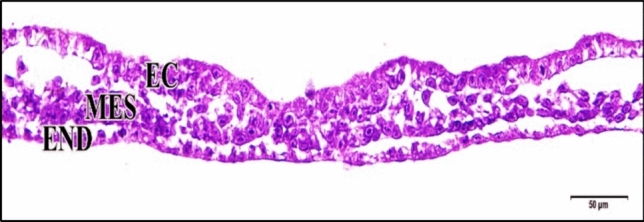
A photomicrograph of a transverse section of a chick embryo treated with 75 µM CdCl_2_ after 33 hours of incubation showing growth retardation. Ectoderm (EC), mesoderm (MES), endoderm (END). H & E stain

The 75 µM CdCl_2_ heart formation was delayed as most embryos revealed growth retardation (Fig. 5).

### At the Embryonic Age of 48 h

**Fig. 6:**
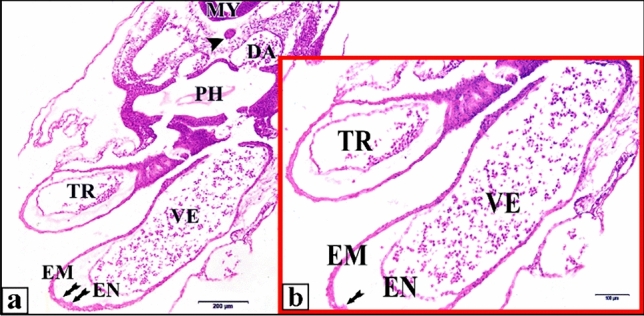
**a** A photomicrograph of a transverse section of a normal heart of 48 hours chick embryo showing its two chambers, truncus arteriosus (TR) and ventricle (VE) and the shallow projections that extended into the cardiac jelly (tailed arrows). **b** A magnification of the two heart chambers. Mylencephalon (MY), notochord (arrow head), dorsal aorta (DA), pharynx (PH), epimyocardium (EM), endocardium (EN). H & E stain

The tubular control or saline heart was completely fused at the level of the presumptive ventricle. The mid-region of the heart is considerably dilated and bent to the right. This bending process is correlated with the rupture of the dorsal mesocardium at the mid-region of the heart. Once the heart tube undergoes looping, the myocardial cells will become contractile. The atria appear as lateral expansions in the primitive atrium region. Continued cardiac looping causes the sinus venosus to move from its original position caudal to the atria towards its definitive position dorsal to the atria. Although there are as yet no sharply bounded subdivisions of the heart, it is convenient to distinguish four regions which later become clearly marked off from each other. So the heart in serial sections seemed as two chambers owing to its looping. At this stage the epimyocardium layer shows irregular shallow projections extended into the cardiac jelly, these projections are the start of trabecular carneae which are conspicuous features of the interior of the adult ventricle (Fig. 6).

**Fig. 7:**
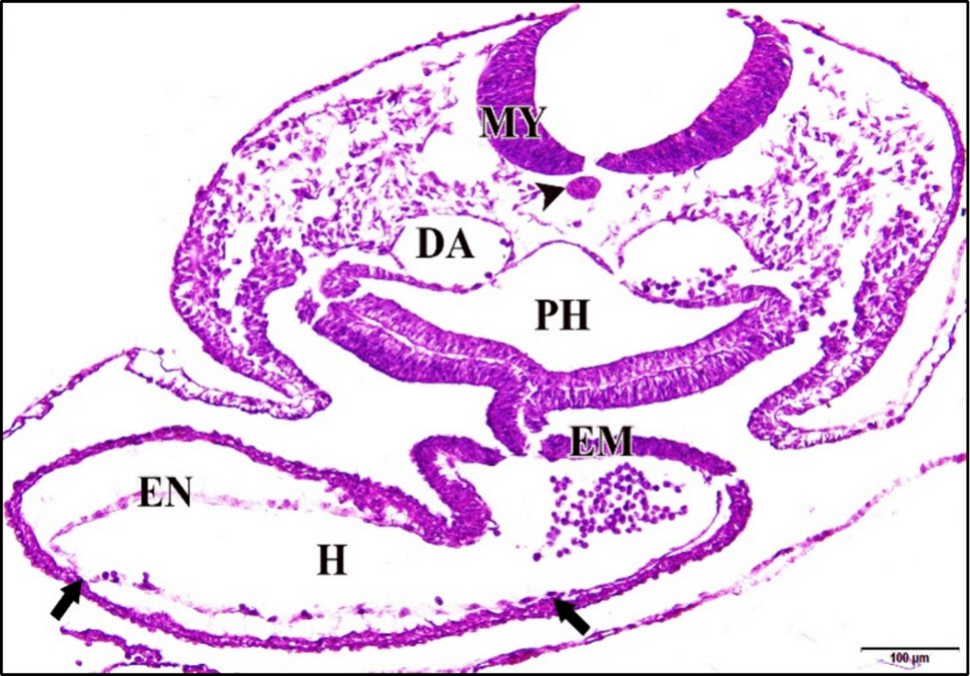
A photomicrograph of a transverse section of a chick embryo treated with 5 µM CdCl_2_ after 48 hours of incubation showing the heart (H) as one chamber, the thickness of epimyocardium (EM) and the connection between the epimyocardium with endocardium (arrows). Mylencephalon (MY), notochord (arrow head), dorsal aorta (DA), pharynx (PH), endocardium (EN). H & E stain

Development rate of 5 µM CdCl_2_ heart was around 15 h behind control. It appeared as one chamber and the epimyocardium was thicker than the control one especially in the dorsal side. Epimyocardium was in contact with endocardium in some regions. Blood cells were seen in the heart lumen (Fig. 7).

**Fig. 8:**
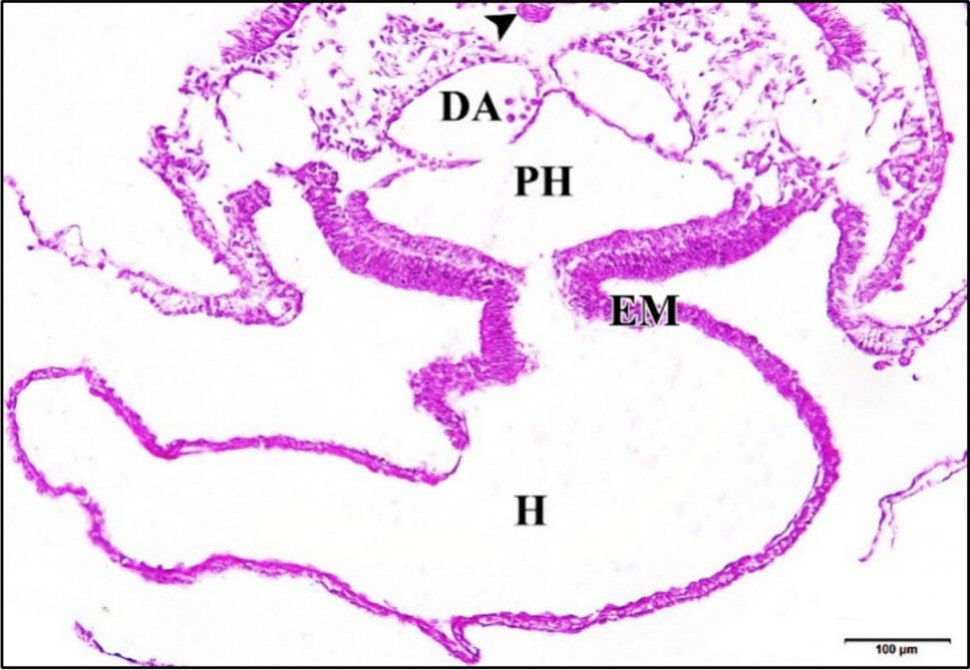
A photomicrograph of a transverse section of a chick embryo treated with 25 µM CdCl_2_ after 48 hours of incubation showing the heart (H) as one chamber and the absence of endocardium and blood corpuscle. Notochord (arrow head), dorsal aorta (DA), pharynx (PH), epimyocardium (EM). H & E stain

Development rate of 25 µM CdCl_2_ heart was eleven to thirteen hours slower than control; where it appeared as one chamber. The endothelium membrane was absent and no blood corpuscles could be observed in the heart cavity. Heart cavity was obviously dilated (Fig. 8).

The 50 µM CdCl_2_ heart showed lateness in growth, where it appeared as one chamber.

**Fig. 9:**
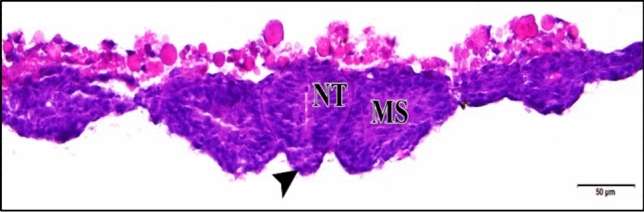
A photomicrograph of a transverse section of a chick embryo treated with 75 µM CdCl_2_ after 48 hours of incubation showing completely deformed embryo. Neural tube (NT), mesodermal somite (MS), notochord (arrow head). H & E stain

Severe malformations were observed in the 75 µM CdCl_2_ embryos. So, the heart was not formed in some cases (Fig. 9).

### At the Embryonic Age of 72 h

**Fig. 10:**
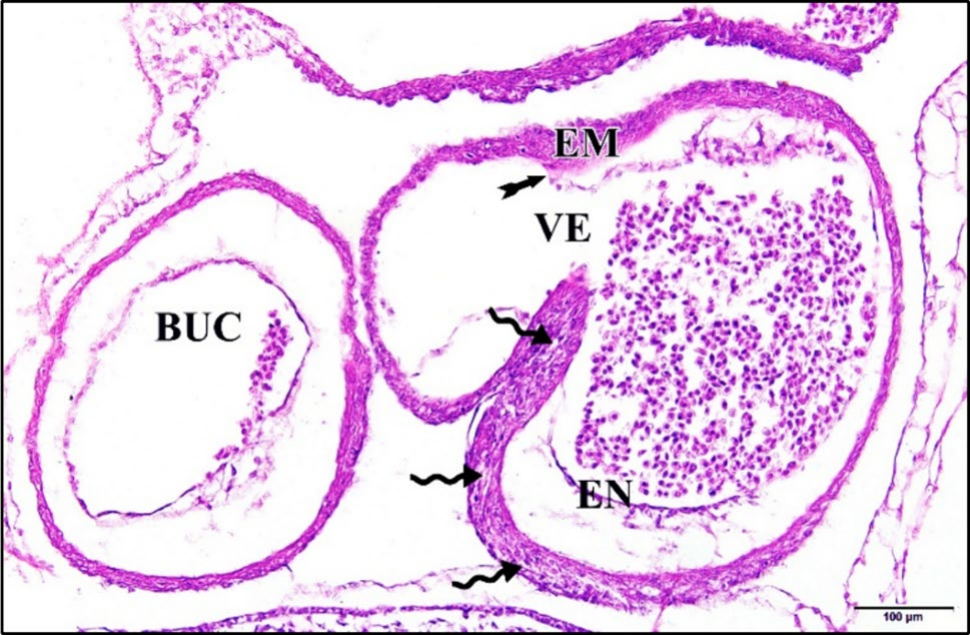
A photomicrograph of a transverse section of a normal heart of 72 hours chick embryo showing the epimyocardium (EM) with clear endomysia between the cardiomyocytes (wavy arrows) and the primordia of trabecular carneae projections (tailed arrow). Endocardium (EN), ventricle (VE), bulb cordis (BUC). H & E stain

**Fig. 11:**
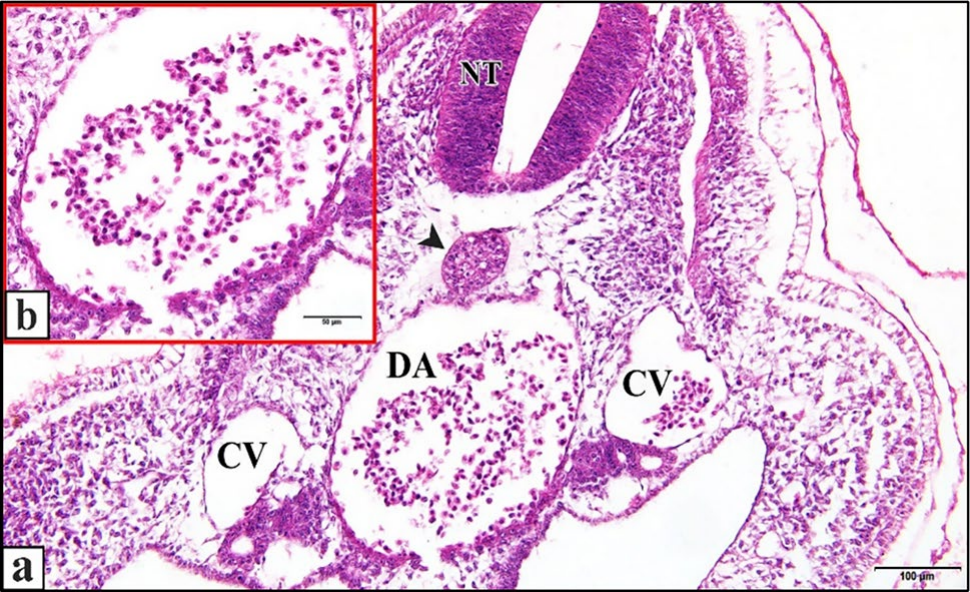
**a **A photomicrograph of a transverse section of a normal chick embryo after 72 hours of incubation through trunk region showing dorsal aorta (DA). **b** A magnification of the dorsal aorta showing its wall and the blood corpuscles inside it. Neural tube (NT), notochord (arrow head), cardinal vein (CV). H & E stain

The epimyocardial layer of the control or saline heart is an outer envelope which surrounds and reinforces the endocardial wall. Cardiomyocytes started to differentiate in the ventral wall of the ventricle. Connection between cardiomyocytes was somewhat loose, where endomysia were clearly seen. The primordia of trabecular carneae are now deeper than before in the cardiac jelly. The endocardium is a single cell layer lining the lumen (Fig. 10). The dorsal aortae fuse in the mid-line to form the unpaired dorsal aorta with blood corpuscles inside (Fig. 11).

**Fig. 12:**
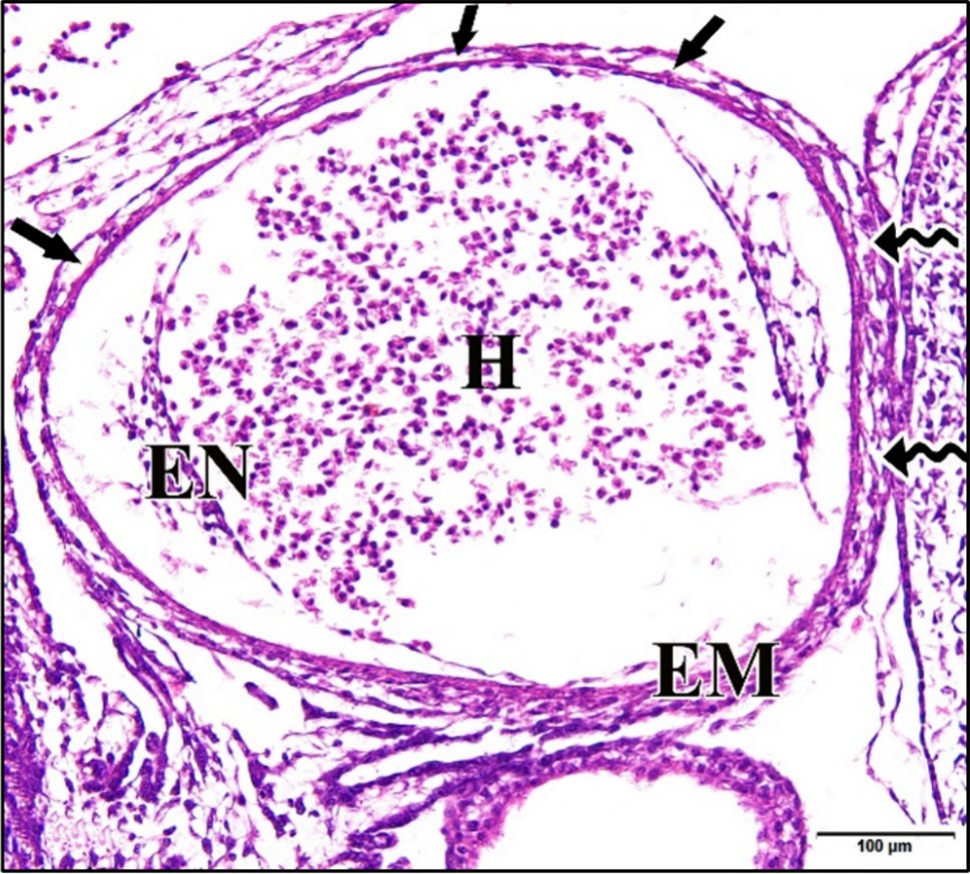
A photomicrograph of a transverse section of a chick embryo treated with 5 µM CdCl_2_ after 72 hours of incubation showing degeneration of epimyocardial (EM) wall with wide endomysia (wavy arrows) and the blood capillaries in the dorsal side of the ventricle (arrows) of the heart (H). Endocardium (EN). H & E stain

**Fig. 13:**
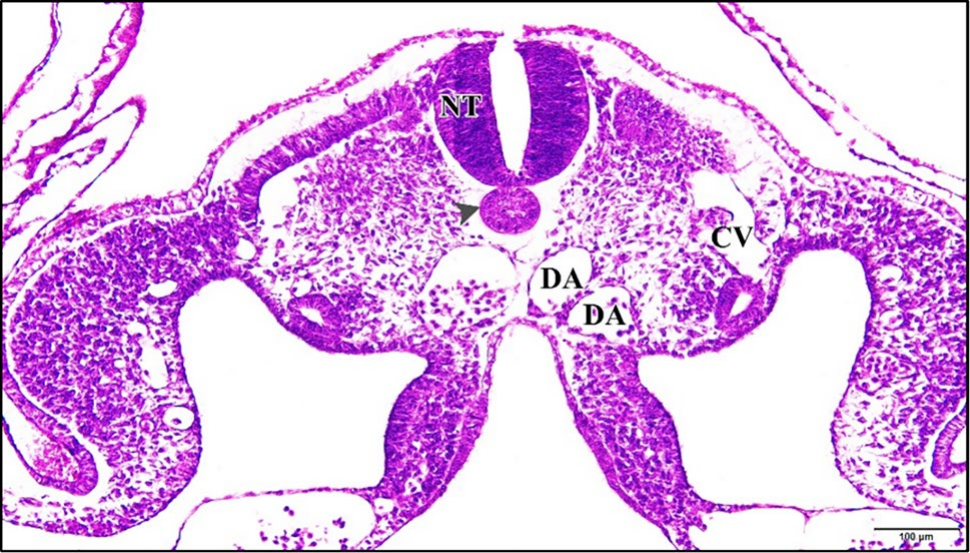
A photomicrograph of a transverse section of a chick embryo treated with 5 µM CdCl_2_ after 72 hours of incubation through trunk region showing separation of dorsal aorta (DA) and its division into two parts. The absence of right posterior cardinal vein (CV). Neural tube (NT), notochord (arrow head). H & E stain

Development rate of the 5 µM CdCl_2_ heart was still few hours behind control, where differentiations of ventricular lumen from the main heart cavity was not observed. The epimyocardial cells were highly degenerated containing endomysia wider than that of the control. Dialated peripheral blood capillaries were obviously observed in the dorsal side of the ventricle (Fig. 12). The two dorsal aortae were still separated from each other and the left dorsal aorta appeared as two parts. Only the left posterior cardinal vein could be observed while the right one was absent (Fig. 13).

**Fig. 14:**
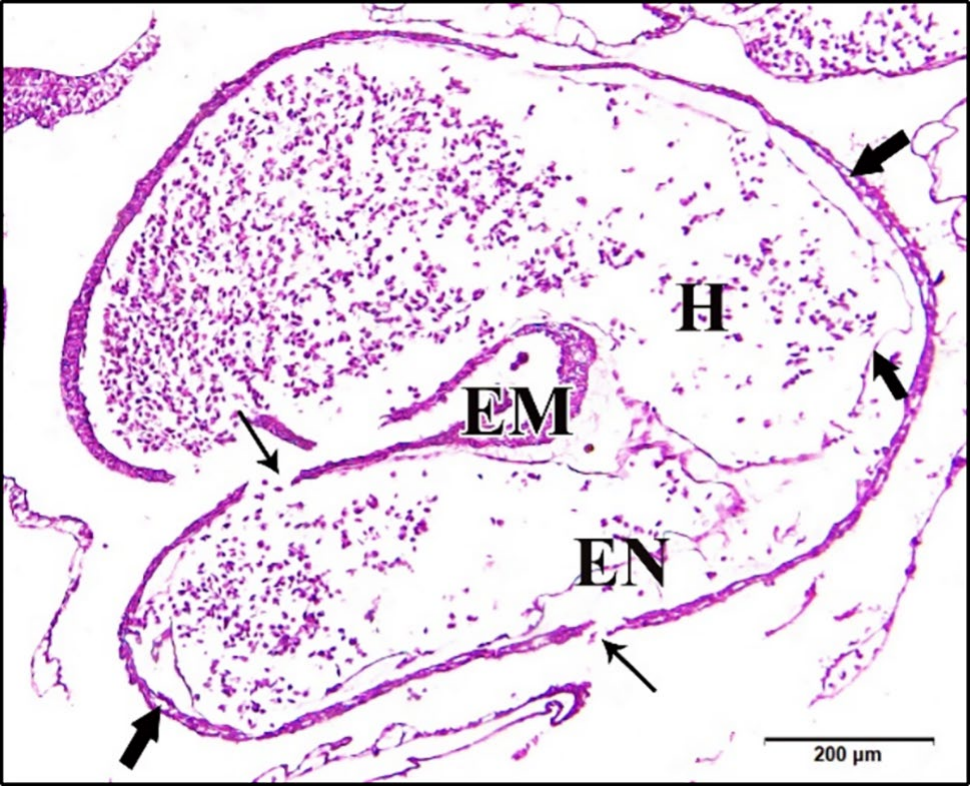
A photomicrograph of a transverse section of a chick embryo treated with 25 µM CdCl_2_ after 72 hours of incubation showing long curved chamber of heart (H) and degeneration of its outer and inner wall (thick arrows) and the ruptures in the thin epimyocardial wall (EM) (thin arrows). Endocardium (EN). H & E stain

**Fig. 15:**
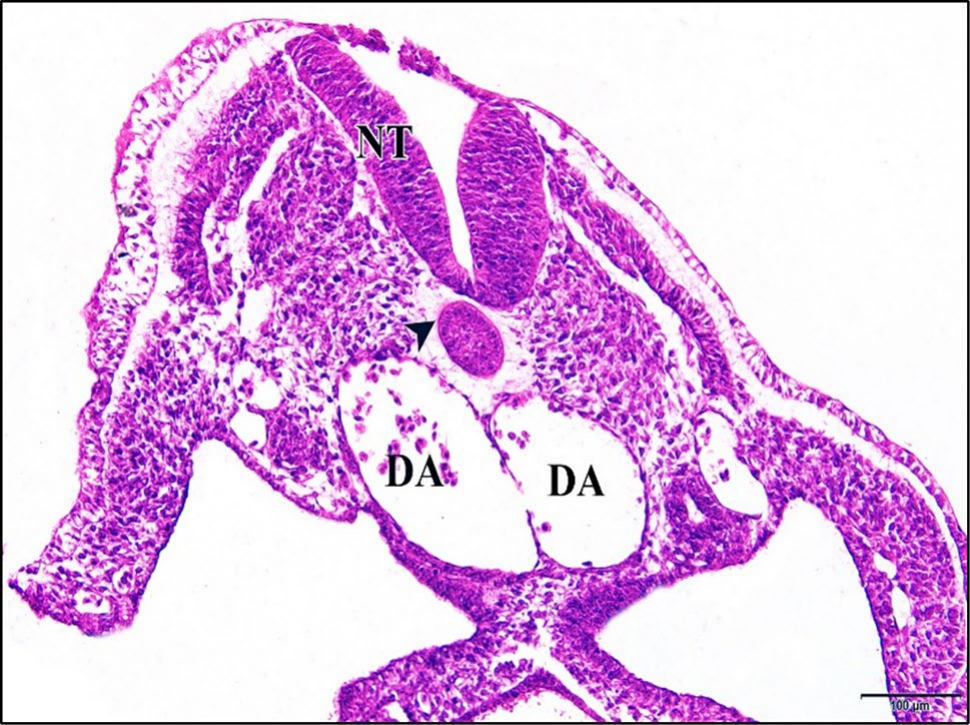
A photomicrograph of a transverse section of a chick embryo treated with 25 µM CdCl_2_ after 72 hours of incubation through trunk region showing unfused dorsal aortae with right big one (DA). Neural tube (NT), notochord (arrow head). H & E stain

The 25 µM CdCl_2_ heart appeared as one long curved chamber with thin degenerated epimyocardium and endocardium. Ruptures in the thin epimyocardial wall were obviously noticed (Fig. 14). The two dorsal aortae were still separated from each other and the right dorsal aorta was bigger than the left one (Fig. 15).

**Fig. 16:**
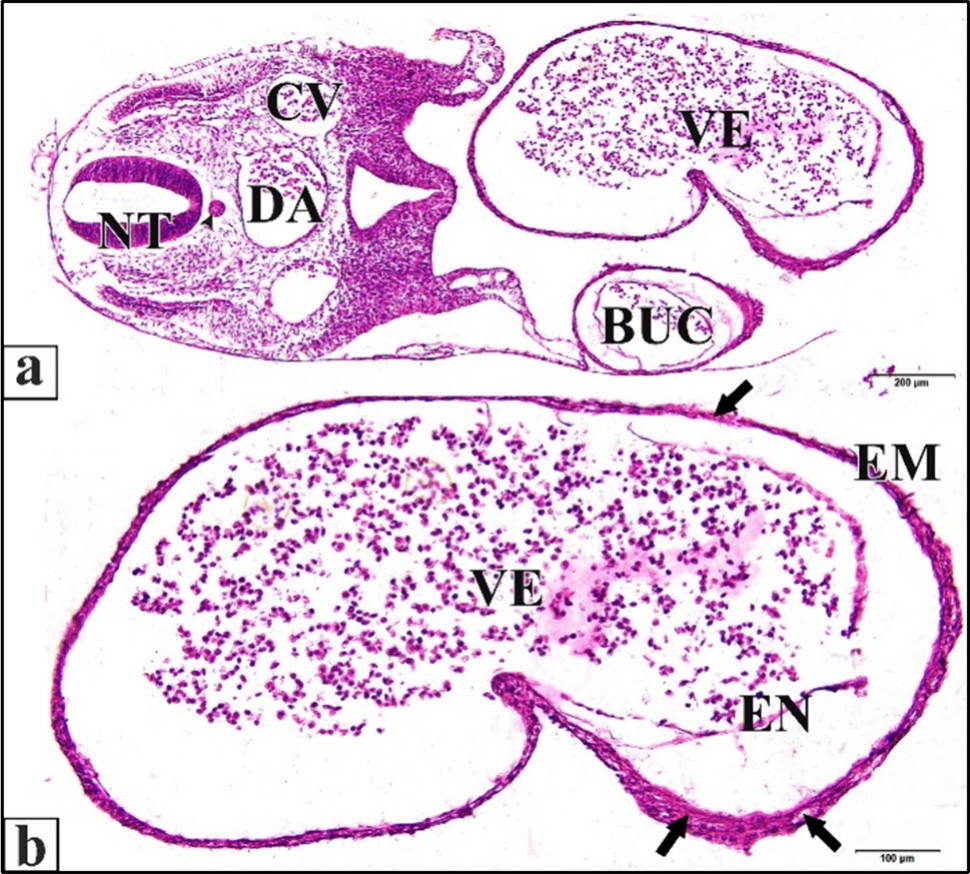
**a **A photomicrograph of a transverse section of a chick embryo treated with 50 µM CdCl_2_ after 72 hours of incubation showing huge curved chamber of heart. **b** A magnification of the ventricle (VE) and degeneration of the epimyocardium wall (EM) with wide endomysia in between (arrows). Neural tube (NT), notochord (arrow head), cardinal vein (CV), dorsal aorta (DA), bulb cordis (BUC), endocardium (EN). H & E stain

**Fig. 17:**
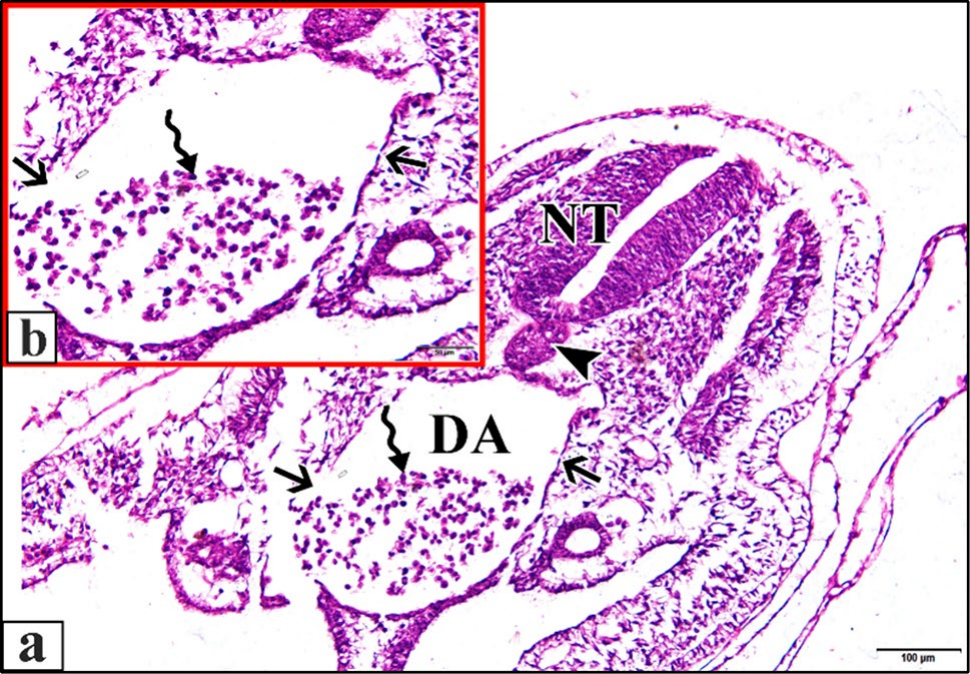
**a** A photomicrograph of a transverse section of a chick embryo treated with 50 µM CdCl_2_ after 72 hours of incubation through trunk region showing degenerated dorsal aorta (DA) with damaged wall (arrows). **b** A magnification of the dorsal aorta showing damage of its wall (arrows) and shrinkage of blood corpuscles with condensed chromatin (wavy arrow). Neural tube (NT), notochord (arrow head). H & E stain

The 50 µM CdCl_2_ heart was huge. Cardiomyocytes of the epimyocardium were loosely connected to each other with obviously wide endomysia in between showing signs of degeneration. Epimyocardium was thinner than control (Fig. 16). Dorsal aorta was degenerated and most of its wall was damaged. Blood corpuscles inside it were shrunk with condensed chromatin (Fig. 17).

**Fig. 18:**
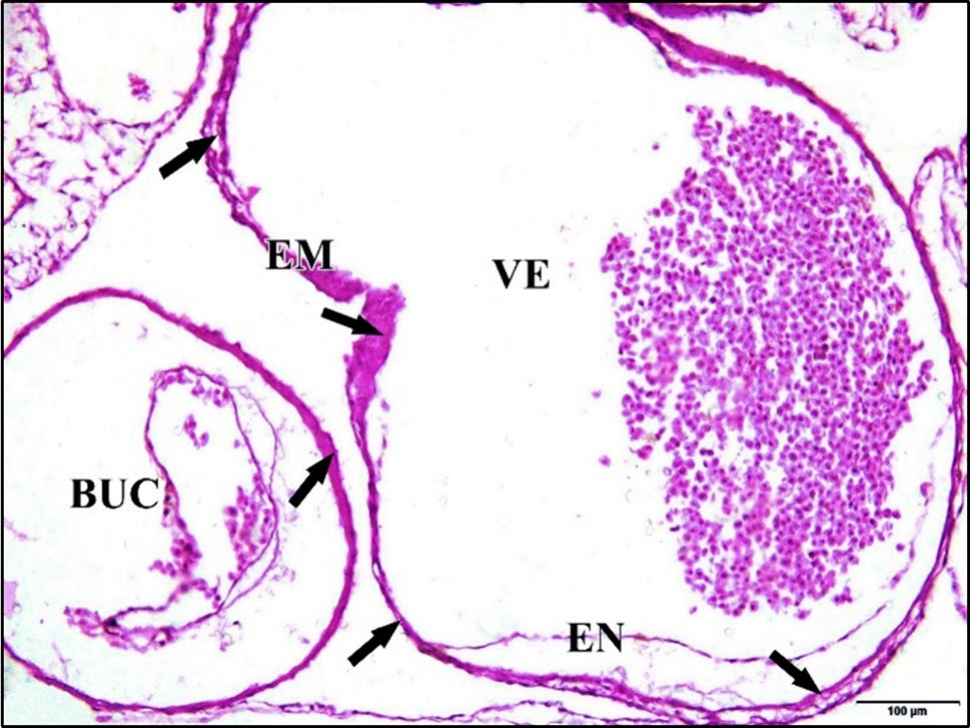
A photomicrograph of a transverse section of a chick embryo treated with 75 µM CdCl_2_ after 72 hours of incubation showing huge heart with thin degraded and vacuolated epimyocardium (EM) (arrows). Ventricle (VE), endocardium (EN), bulb cordis (BUC). H & E stain

**Fig. 19:**
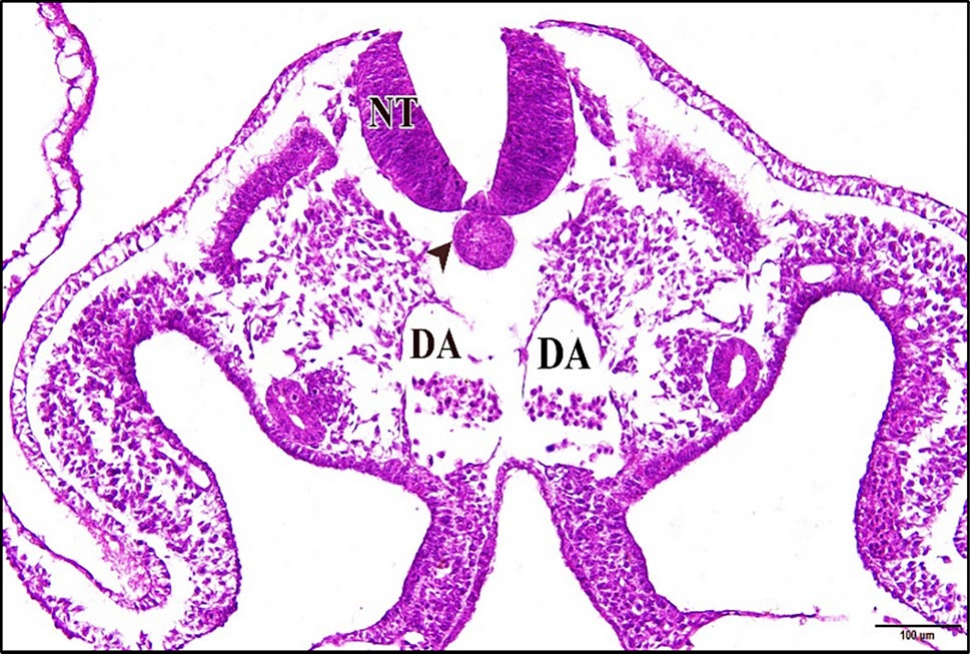
A photomicrograph of a transverse section of a chick embryo treated with 75 µM CdCl_2_ after 72 hours of incubation through trunk region showing separated and broken dorsal aortae (DA). Neural tube (NT), notochord (arrow head). H & E stain

The 75 µM CdCl_2_ heart was megascopic than control and its wall was thin, degenerated and vacuolated (Fig. 18). Dorsal aorta was still appearing as two separated and broken vessels (Fig. 19).

### At the Embryonic Age of 96 h

**Fig. 20:**
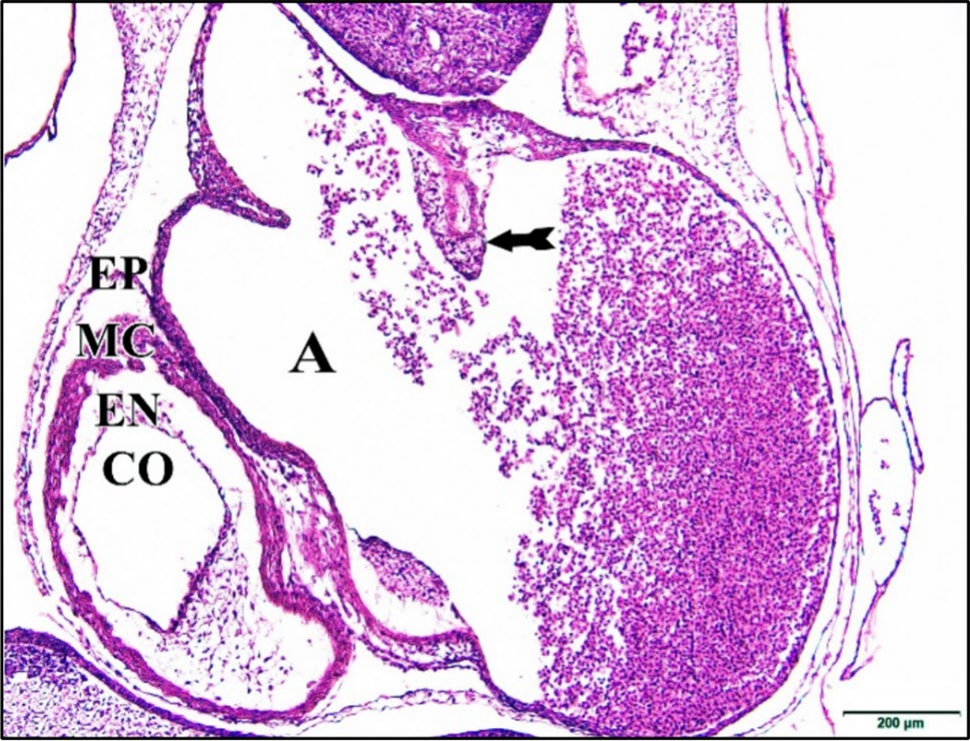
A photomicrograph of a transverse section of a normal heart of 96 hours chick embryo showing single cell layered endocardium (EN) lining heart lumen and thickened epimyocardium and its differentiation into myocardium (MC) and epicardium (EP) and the growing and extensions of the irregular projections from the myocardial layer into the cardiac jelly (tailed arrow). Atrium (A), conus arteriosus (CO). H & E stain

General growth of the control or saline heart and considerable expansion of the ventricular bend and the primitive atria have led to the loss of the original tubular character of the heart. As development progresses the original epimyocardium in the heart of a four-day chick becomes greatly thickened and is finally can be differentiated into two layers, an inner heavy muscular layer, myocardial layer (myocardium) and an outer thin non-muscular covering layer, epicardial layer (epicardium). The endocardium is still a single cell layer lining the lumen. As the irregular projections (trabeculae) that extended from the myocardial layer into the cardiac jelly grow, the endocardium lining tends to surround them (lined intertrabecular spaces). The endocardium follow closely the configuration of the myocardial trabecular separated from them by a thin layer of cardiac jelly (Fig. 20).

**Fig. 21:**
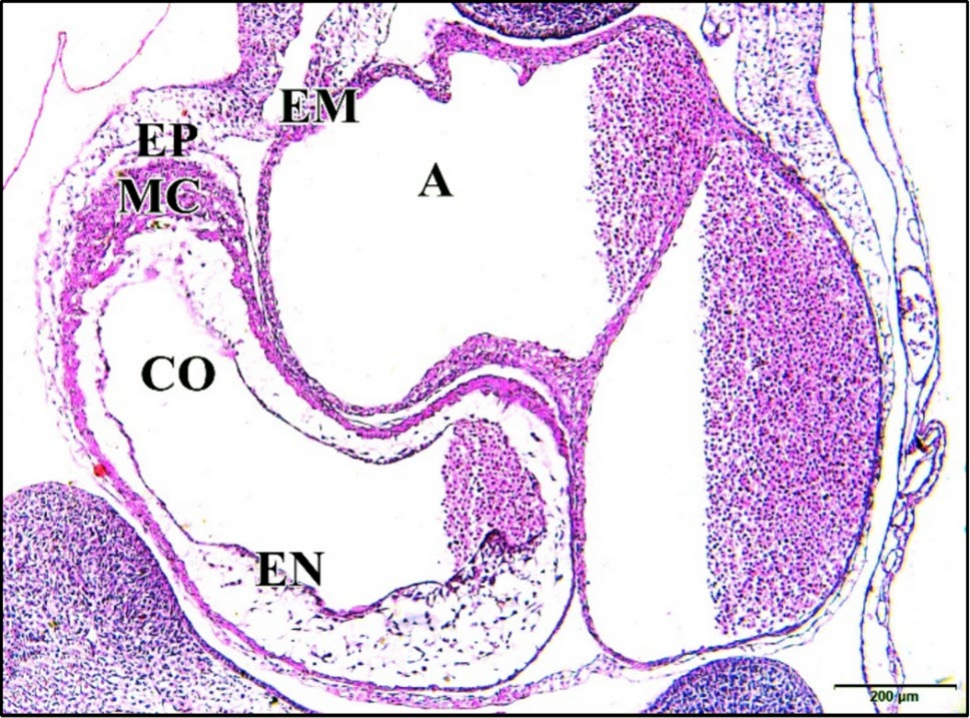
A photomicrograph of a transverse section of a chick embryo treated with 25 µM CdCl_2_ after 96 hours of incubation showing a huge heart. Atrium (A), conus arteriosus (CO), epimyocardium (EM), epicardium (EP), myocardium (MC), endocardium (EN). H & E stain

The 5 & 25 µM CdCl_2_ hearts were very huge in size comparing with the normal one (Fig. 21).

**Fig. 22:**
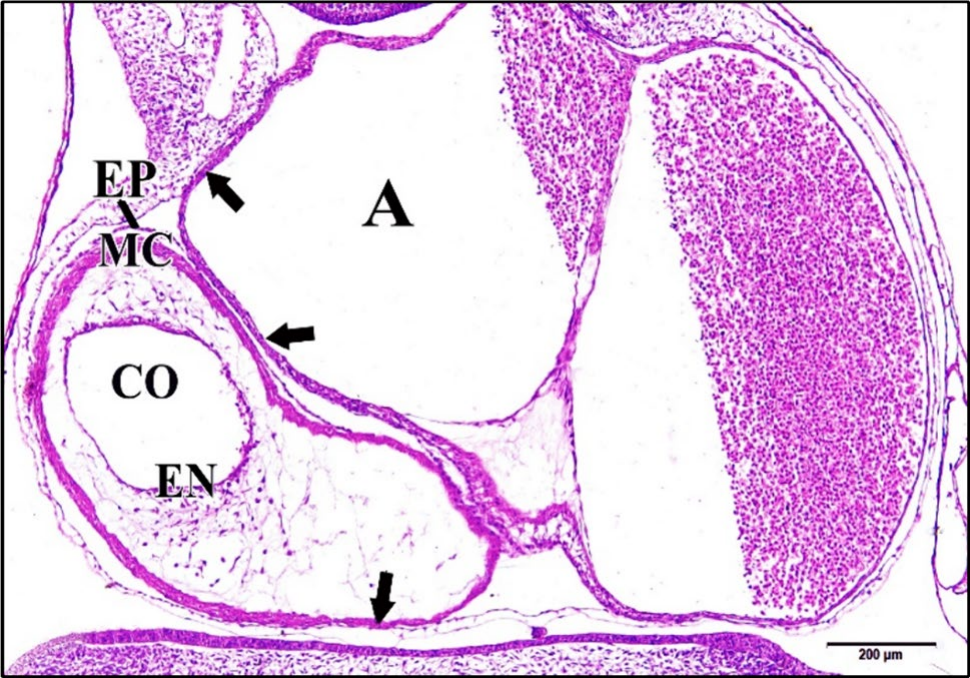
A photomicrograph of a transverse section of a chick embryo treated with 75 µM CdCl_2_ after 96 hours of incubation showing enlarged heart with thin myocardium (MC) (arrows). Atrium (A), conus arteriosus (CO), epicardium (EP), endocardium (EN). H & E stain

In general, the 50 & 75 µM CdCl_2_ hearts appeared large and huge in size, where its cavity were wider than that of the control one. Epicardium was hardly observed in the former one and the myocardium was thinner than that of the control (Fig. 22).

### At the Embryonic Age of 144 h

From embryonic day six to embryonic day eight in the control cases, there is an 80% increase in cell density in the heart ventricular myocardial compact zone. The walls of the atria and ventricles are similar in organization, although the walls are thicker in the ventricles. The heart walls contain three distinct layers, the inner endocardium, the middle myocardium and the outer epicardium.

**Fig. 23:**
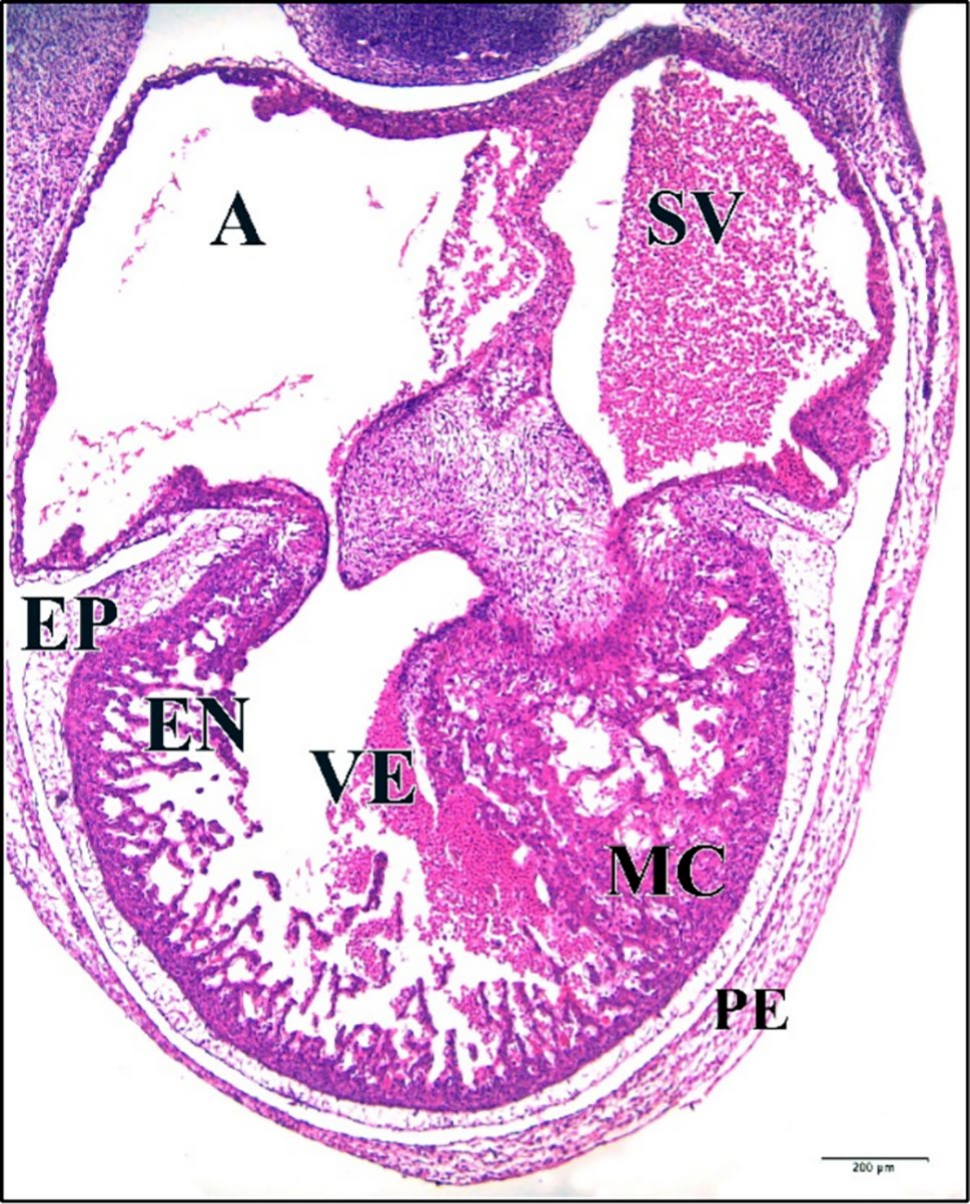
A photomicrograph of a transverse section of a normal heart of 144 hours chick embryo showing increase in cell density in the heart ventricular myocardial compact zone, elongation of its cells characterizing the developing muscle cells and their arrangement in bundles projecting into heart lumen. Atrium (A), sinus venosus (SV), ventricle (VE), pericardium (PE), epicardium (EP), myocardium (MC), endocardium (EN). H & E stain

The endocardium, which surrounds the lumen of the heart. The thickest layer of the heart is the myocardium, which is composed of cardiomyocytes. The myocardium has become greatly thickened and the cells in it are elongated and began to show the histological characteristics of developing myocytes. Their arrangement in bundles which project toward the lumen fore-shadows the formation of the muscle bands (trabeculae carneae) which ridge the inner wall of the adult heart. The third and outer layer of the heart is the epicardium, which contains connective tissue with elastic fibers, adipose tissue and nerves, as well as large coronary arteries and veins. The epicardium is covered by a mesothelium, the visceral pericardium. Although it is not considered a separate layer, the heart lies in the pericardium (Fig. 23).

**Fig. 24:**
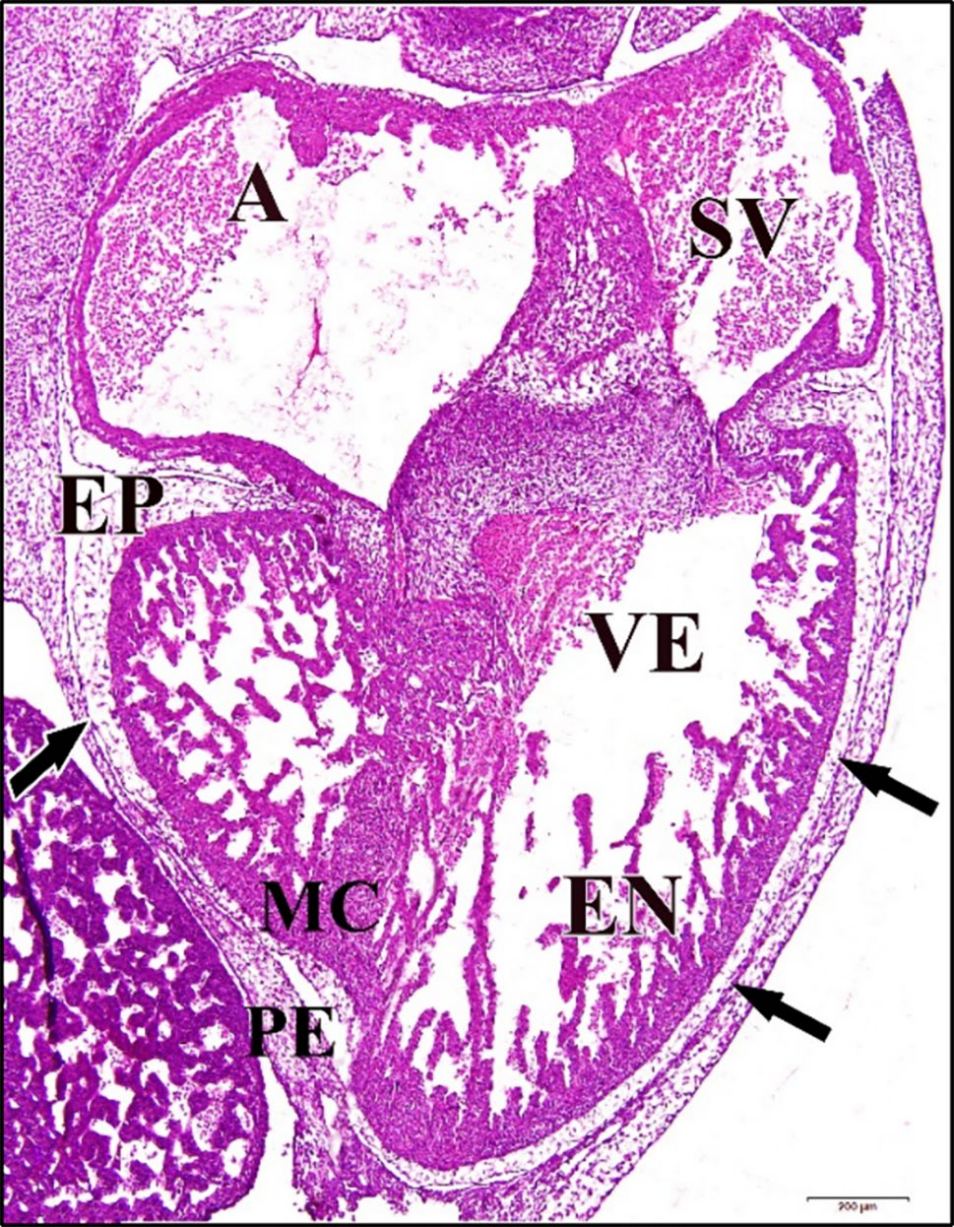
A photomicrograph of a transverse section of a chick embryo treated with 5 µM CdCl_2_ after 144 hours of incubation showing small chambers of heart, atrium (A) and sinus venosus (SV), and disappearance of the space between heart external membranes pericardium (PE) and epicardium (EP) (arrows). Ventricle (VE), myocardium (MC), endocardium (EN). H & E stain

Atrium and sinus venosus of the 5 µM CdCl_2_ heart appeared small in size compared to control. Space between pericardium and epicardium layers disappeared in some regions (Fig. 24).

**Fig. 25:**
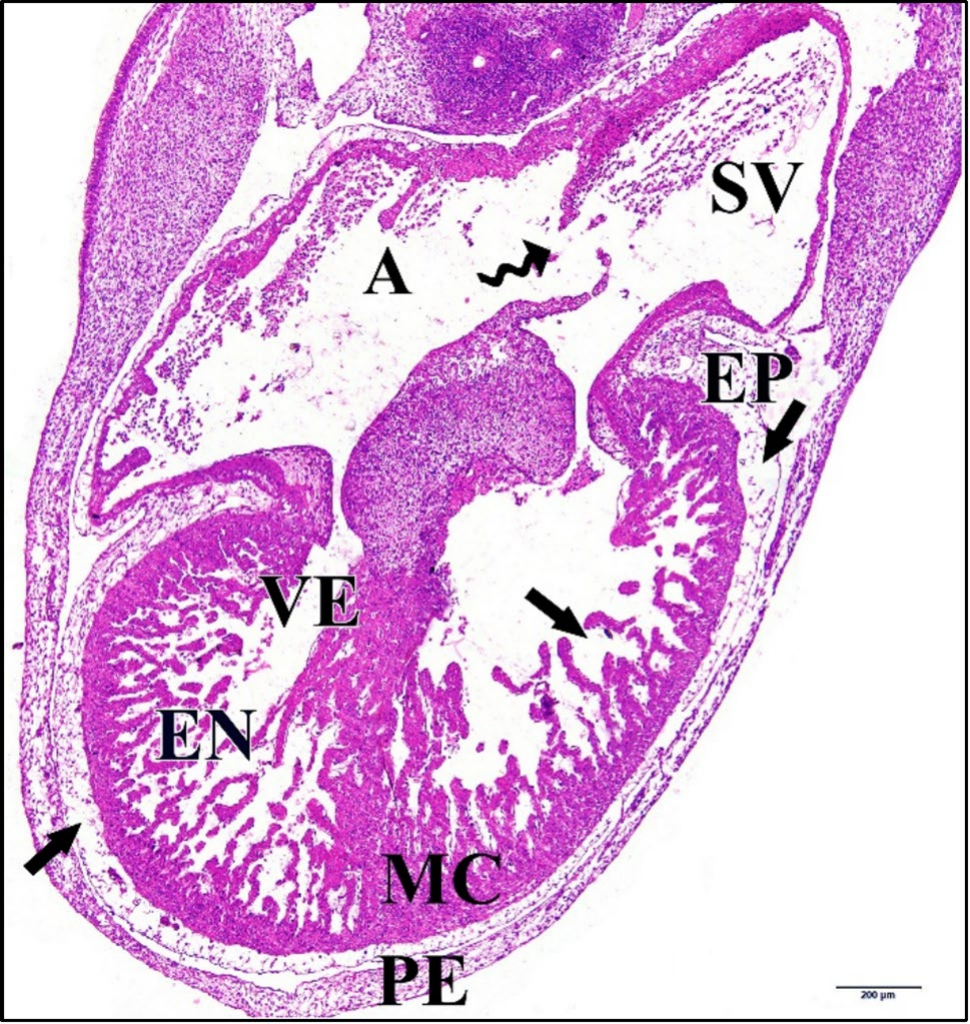
A photomicrograph of a transverse section of a chick embryo treated with 25 µM CdCl_2_ after 144 hours of incubation showing large heart with degeneration and vacuolations of its walls (arrows) and the ruptures of the wall that separating atrium (A) and sinus venosus (SV) (wavy arrow). Ventricle (VE), pericardium (PE), epicardium (EP), myocardium (MC), endocardium (EN). H & E stain

The 25 µM CdCl_2_ heart was large. The wall separating atrium and sinus venosus was ruptured. Atrial and ventricular walls are thinner than the normal, especially epicardium which was highly vacuolated and degenerated (Fig. 25).

**Fig. 26:**
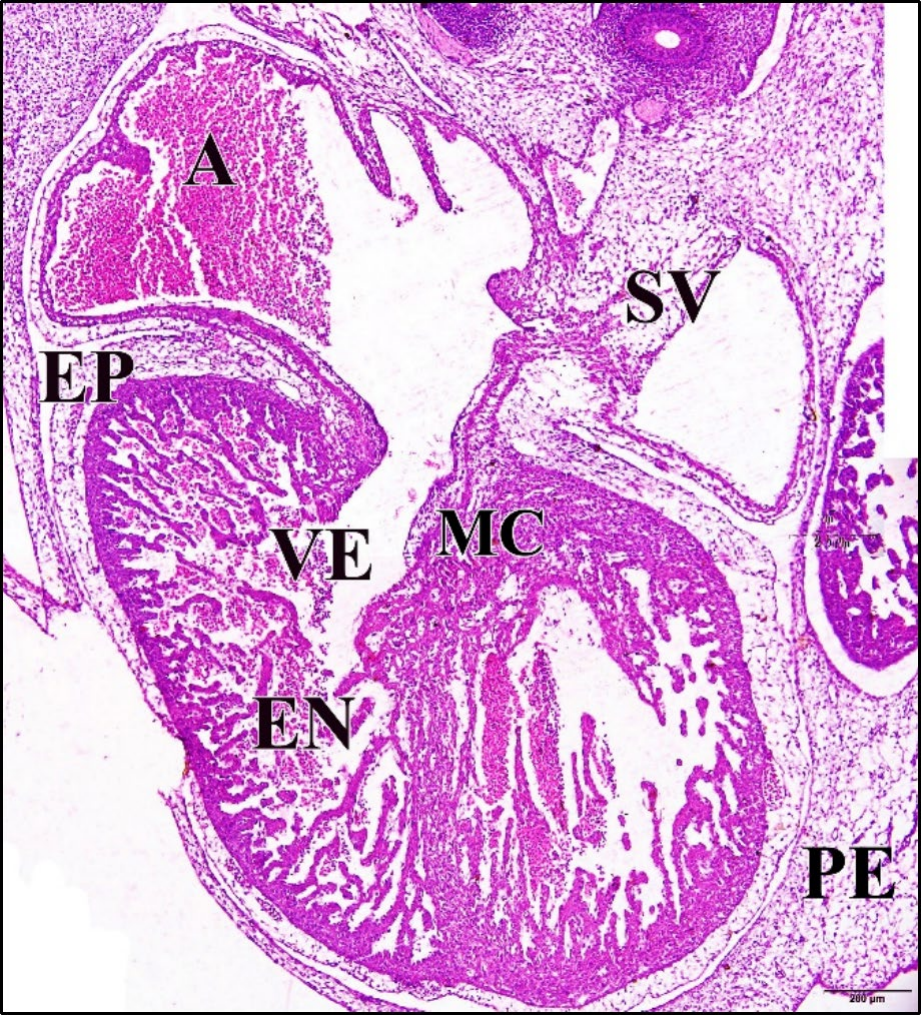
A photomicrograph of a transverse section of a chick embryo treated with 50 µM CdCl_2_ after 144 hours of incubation showing small heart chambers. Atrium (A), sinus venosus (SV), ventricle (VE), pericardium (PE), epicardium (EP), myocardium (MC), endocardium (EN). H & E stain

The 50 µM CdCl_2_ heart chamber lumens were small comparing with the control (Fig. 26).

**Fig. 27:**
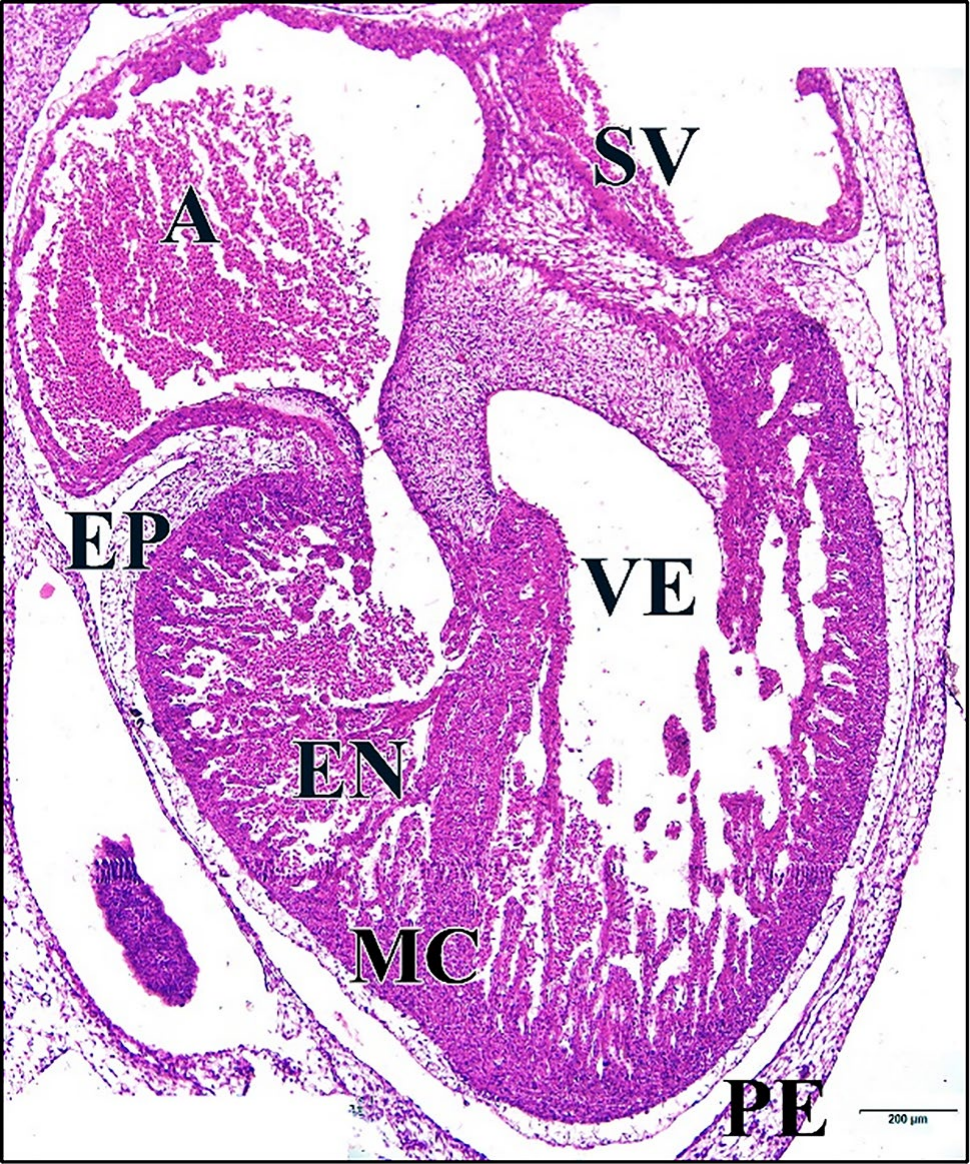
A photomicrographs of a transverse section of a chick embryo treated with 75 µM CdCl_2_ after 144 hours of incubation showing large heart. Atrium (A), sinus venosus (SV), ventricle (VE), pericardium (PE), epicardium (EP), myocardium (MC), endocardium (EN). H & E stain

The 75 µM CdCl_2_ heart was relatively large (Fig. 27).

### After Hatching (21 Days)

**Fig. 28:**
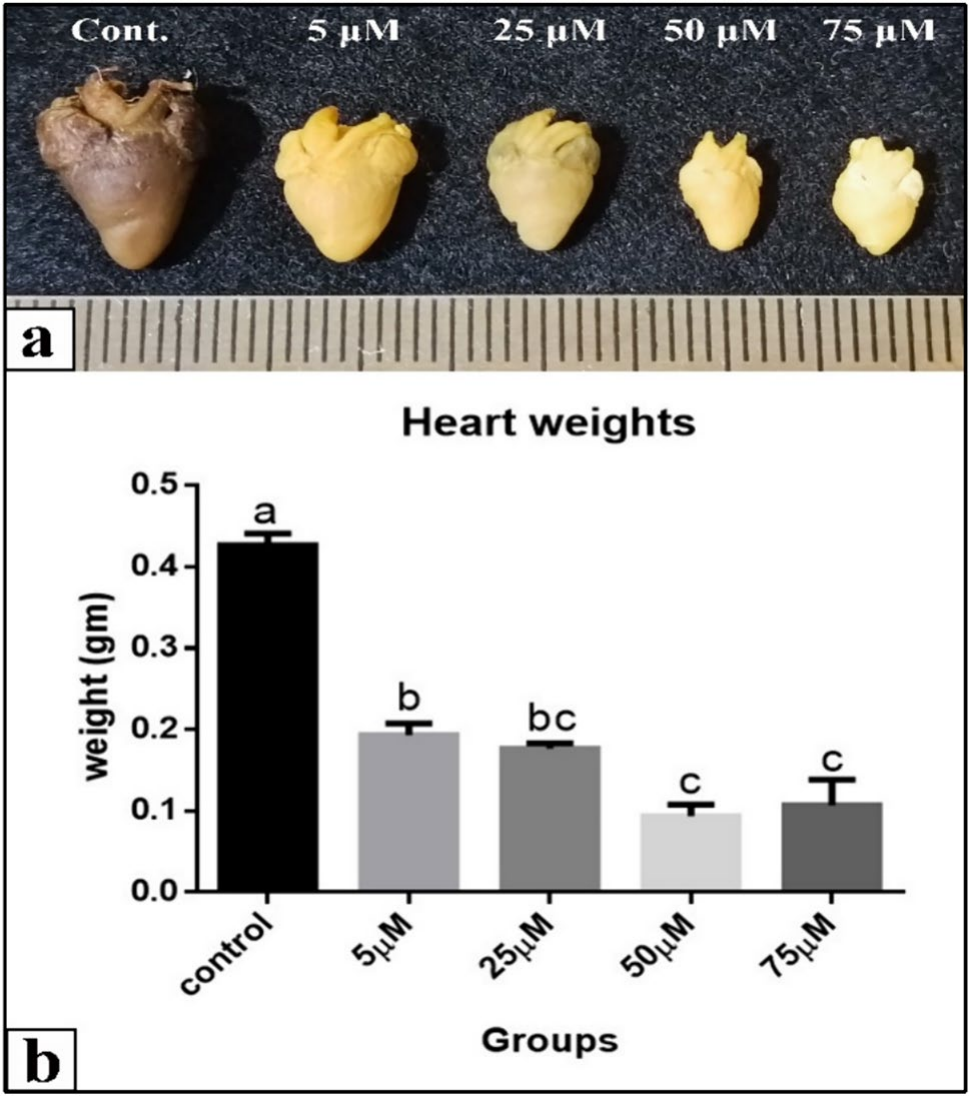
**a **A photograph of heart in all groups at 21 days (after hatching) of incubation showing a comparison of heart size and weight among different groups. **b** Heart weight measurements (gm). A comparison between different treated groups, at 21 days (newly hatching). (a, b, c): significant difference comparing to control

Malformations that Cd induced were obvious in the morphology of heart in all Cd groups compared with the normal one. Heart in all Cd groups was small in size, especially in 50 & 75 µM treatments which showed the heart smaller in size compared with the control and the lower doses. Measurements of the heart weight revealed a decrease in all treated groups compared to the control. Statistical analysis indicated that treatment with Cd (5, 25, 50 & 75 µM) resulted in highly significant decrease (0.1933, 0.1767, 0.0933 and 0.1067 gm respectively) of the heart weight compared to control (0.4267 gm), where *P* < 0.001. Among treated groups, the lowest dose (5 µM) was significantly different comparing to the two highest doses (50 & 75 µM) Cd treated groups, where *P* < 0.05 reflecting dose dependent effect of CD on heart weight (Fig. 28).

**Fig. 29:**
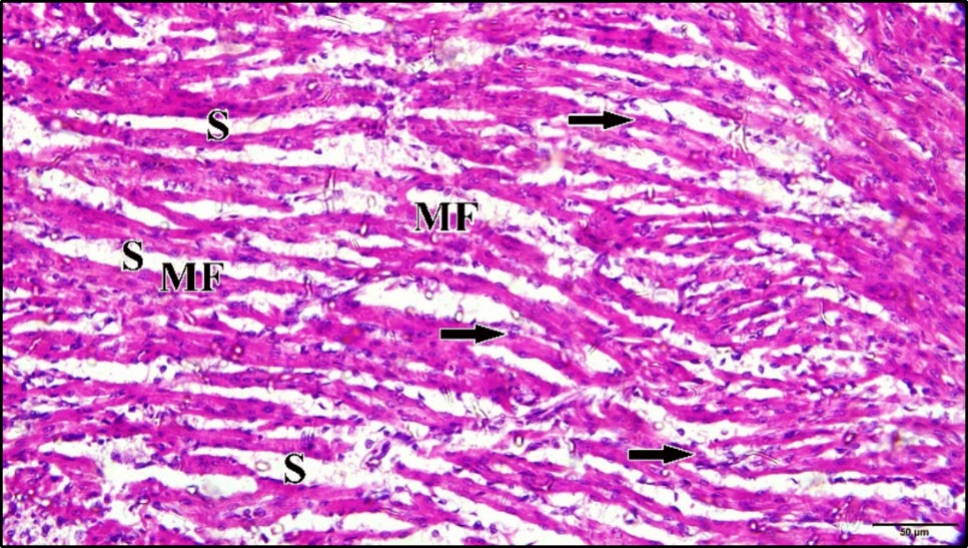
A longitudinal section of a normal cardiac muscles in a chick embryo at 21 days of incubation (after hatching) shows striated, branching and anastomosing muscle fibers (MF) with centrally located oval vesicular nuclei (arrow). The intercellular spaces; endomysia (S) were noticed between myocardial fibers. H & E stain

Microscopic examination of the longitudinal sections of the control or saline heart tissues revealed that heart is composed of cardiac muscle, the myocardium, which was formed of a continuous network of elongated bands of muscle fibers with interposed oval nuclei (Fig. 29).

**Fig. 30:**
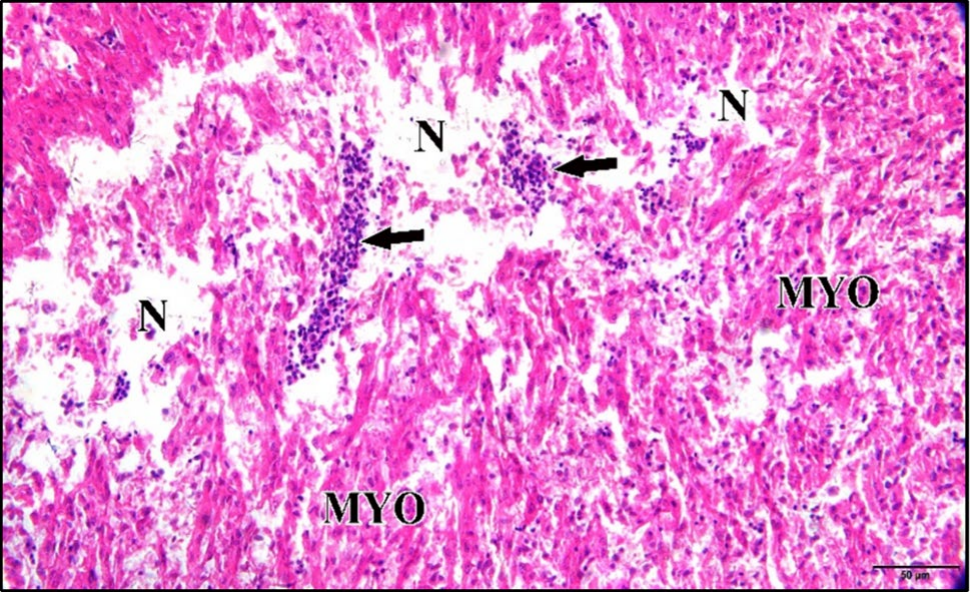
A photomicrograph of a longitudinal section of cardiac muscles in a chick embryo treated with 5 µM CdCl_2_ at 21 days of incubation (after hatching) showing, losing of architecture of cardiac muscles, severe necrosis (N) and myolysis (MYO) signs in the majority of cardiac muscle cells. Also, inflammatory cells infiltration (arrow) were observed. H & E stain

Microscopic inspection of the 5 µM CdCl_2_ heart revealed that most of embryos injected with this dose showed losing of architecture of cardiac muscles. Also it revealed severe necrosis and myolysis with inflammatory cells infiltration in between the fragmented muscle fibers (Fig. 30).

**Fig. 31:**
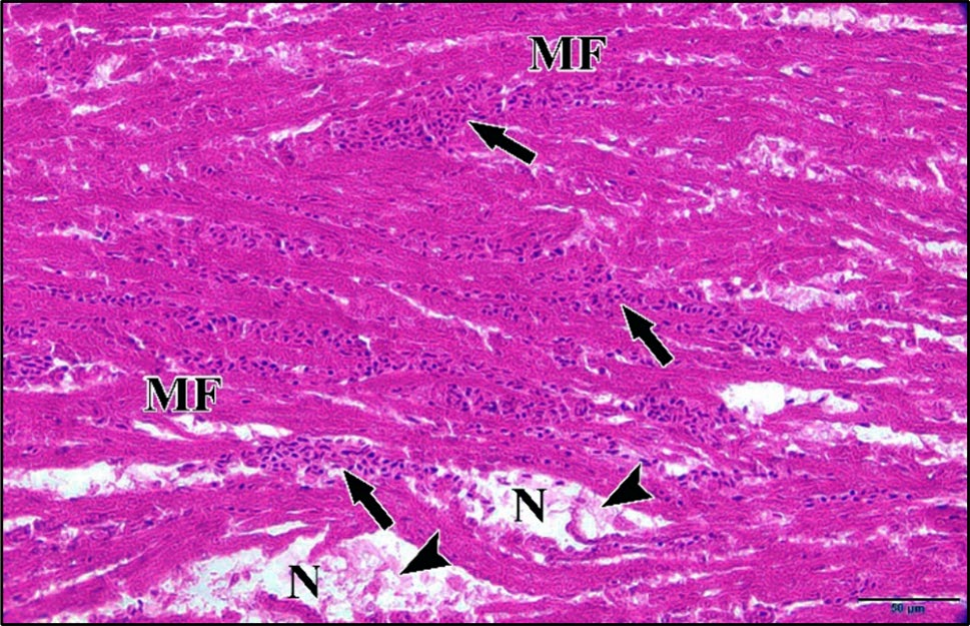
A photomicrograph of a longitudinal section of cardiac muscles in a chick embryo treated with 25 µM CdCl_2_ at 21 days of incubation (after hatching) revealing hypertrophic cardiac muscle fibers (MF), necrotic appearance (N). Also, marked inflammatory cells infiltration (arrow) and edema (arrow head) were noticed. H & E stain

Microscopic examination of the myocardial tissue sections of the embryos injected with 25 µM Cd revealed hypertrophic cardiac muscle fibers, necrosis and edema. Also inflammatory changes indicative of interstitial myocarditis. Muscle fibers were spaced apart, with a large inflammatory infiltration of mononuclear cells (mainly lymphocytes) in between (Fig. 31).

**Fig. 32:**
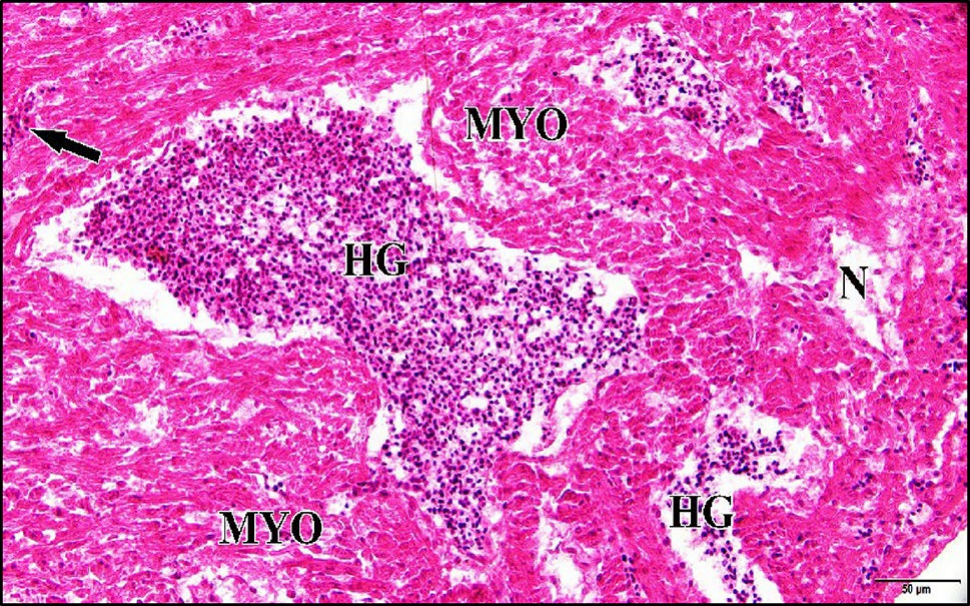
A photomicrograph of a longitudinal section of cardiac muscles in a chick embryo treated with 50 µM CdCl_2_ at 21 days of incubation (after hatching) displaying myolysis (MYO) signs in the majority of cardiac muscle cells with huge hemorrhage (HG) in between them. Also necrotic appearance (N) and marked inflammatory cells infiltration (arrow) were noticed. H & E stain

The microscopic investigation of the myocardial tissue sections obtained from embryos injected with 50 µM Cd showed myolysis of cardiac muscle fibers with huge hemorrhage and necrosis. Also inflammatory cells infiltration were detected (Fig. 32).

**Fig. 33:**
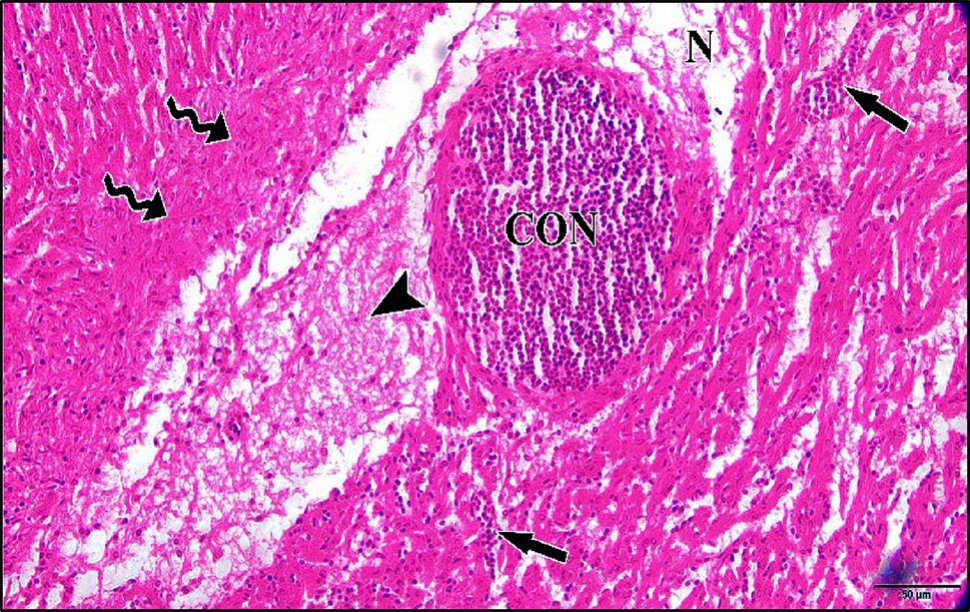
A photomicrograph of a longitudinal section of cardiac muscles in a chick embryo treated with 75 µM CdCl_2_ at 21 days of incubation (after hatching) exhibiting necrosis (N) with disfiguration of muscles architecture (wavy arrows) and remarkable congestion (CON) of blood vessels with inflammatory cells infiltration (arrows) and huge edema (arrow head). H & E stain

Microscopic observation of the myocardial tissue sections obtained from embryos injected with 75 µM Cd displayed marked congestion of dilated blood vessels whose disrupted endothelial lining cells with noticeable edema. Also, necrosis of muscle fibers and disfiguration of muscles architecture with inflammatory cells infiltration were viewed (Fig. 33).

### Morphometric Measurements of Heart

#### Truncus Arteriosus

**Fig. 34:**
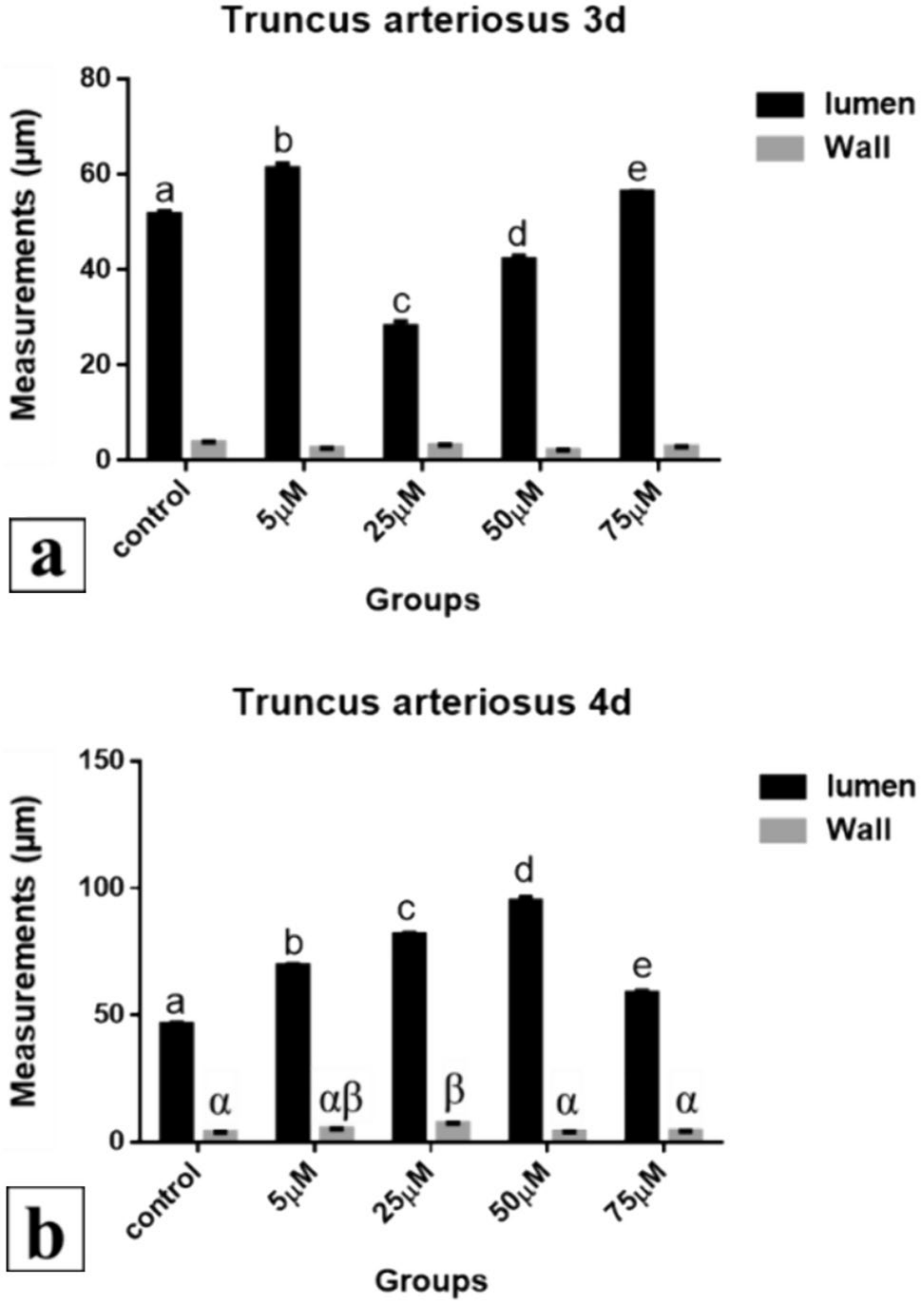
Truncus arteriosus measurements (µm). A comparison between different treated groups, **a** 72 hours after treatment, **b** 96 hours after treatment. (a, b, c, d, e) & (α, β): significant difference comparing to control

*After 72 h of incubation*, 5 and 75 µM Cd treated hearts showed significantly increased (61.252 and 56.288 μm respectively) lumen of truncus arteriosus but such lumen was significantly decreased (28.113 and 42.170 μm) in 25 and 50 µM treated ones respectively compared to control (51.559 μm) (*P* < 0.001). All treatments revealed no significant decrease (2.542, 3.178, 2.092 and 2.847 μm respectively) in the wall of truncus arteriosus compared to control (3.780 μm) (*P* < 0.05) (Fig. 34a).

*After 96 h of incubation*, all the Cd treated groups revealed a significant increase (69.498, 81.539, 94.927 and 58.510 μm respectively) in the lumen of truncus arteriosus compared to the control group (46.318 μm) and to each other (*P* < 0.001). The thickness of truncus arteriosus wall was insignificantly increased (5.211, 3.871 and 4.097 μm) in 5, 50 and 75 µM Cd treated groups respectively (*P* < 0.05). 25 µM group showed significant increase (7.367 μm) compared to the control (3.673 μm) (*P* < 0.01) (Fig. 34b).

### Ventricle

**Fig. 35:**
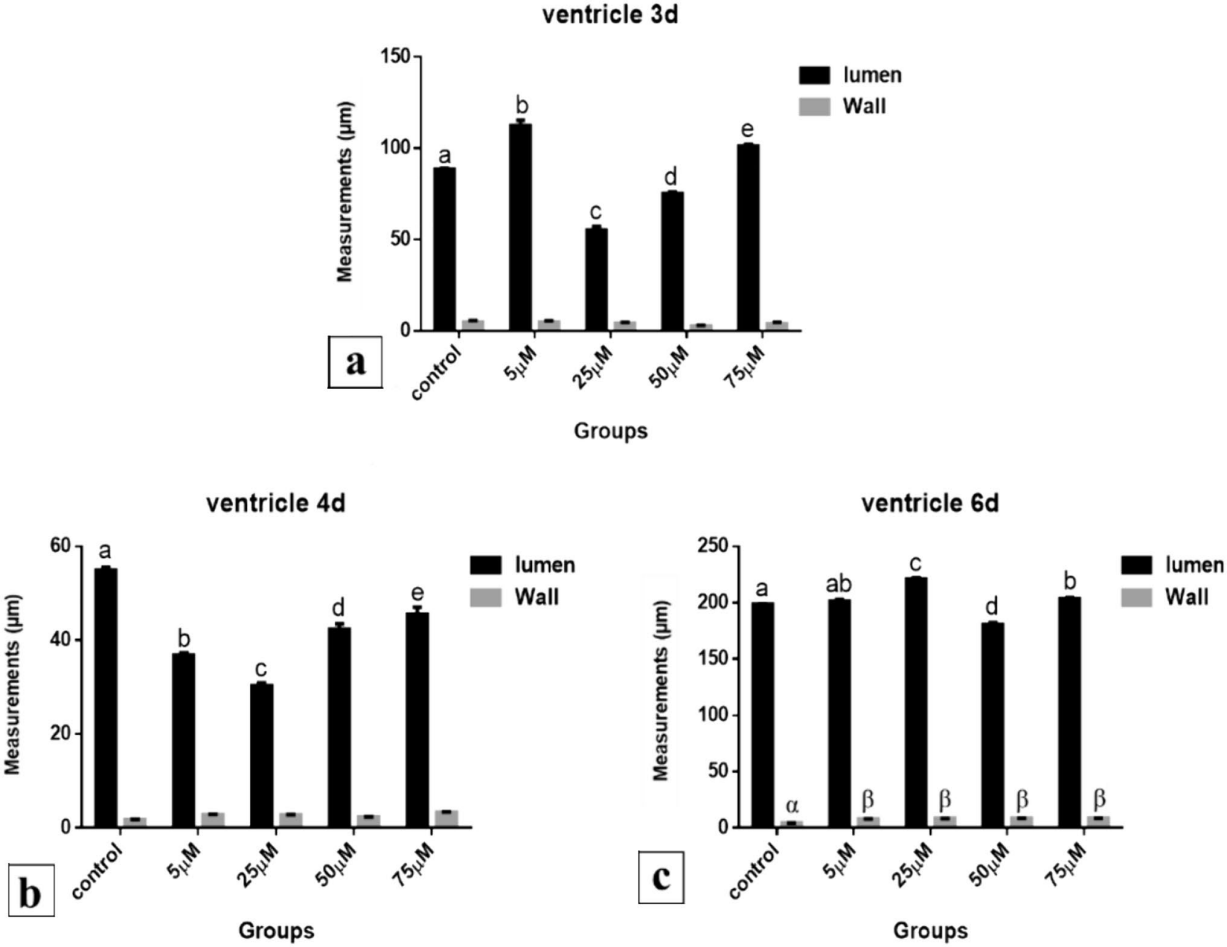
Ventricle measurements (µm). A comparison between different treated groups, **a** 72 hours after treatment, **b** 96 hours after treatment, **c** 144 hours after treatment. (a, b, c, d, e) & (α, β): significant difference comparing to control

*After 72 h of incubation*, the lumen of ventricle was significantly increased (112.496 and 101.162 μm) in 5 and 75 µM Cd treated groups respectively, while in 25 and 50 µM Cd treated groups the lumen was significantly decreased in size (55.255 and 75.336 μm respectively) compared to the control (88.382 μm) (*P* < 0.001). The ventricle wall thickness was insignificantly changed, where in the 5, 25, 50 and 75 µM groups it was (4.861, 4.181, 2.657 and 3.880 μm respectively) compared to control (4.849 μm) (*P* < 0.05) (Fig. 35a).

*After 96 h of incubation*, lumen of ventricle in all Cd treated groups was highly significant decreased in its size (36.804, 30.337, 42.311 and 45.578 μm respectively) compared to control (54.908 μm) (*P* < 0.001). Also, the decrease in the lumen size of ventricle between the 5 and 25 µM doses was highly significant compared to the other two doses 50 and 75 µM Cd treated groups (*P* < 0.001). The morphometric measurements of the ventricle wall thickness showed insignificant increase (2.759, 2.719, 2.251 and 3.348 μm respectively) in all treatments compared to control (1.761 μm) (*P* < 0.05) (Fig. 35b).

*After 144 h of incubation*, the lumen size of ventricle was insignificantly increased (201.413 μm) in 5 µM Cd treated group (*P* < 0.05) and significantly increased (220.923 and 203.742 μm) in the 25 and 75 µM doses of Cd respectively, while it was significantly decreased (180.365 μm) in the Cd treated group with 50 µM Cd compared to control (198.560 μm) (*P* < 0.001). The thickness of the ventricle wall was significantly increased (7.901, 8.152, 8.499 and 8.576 μm respectively) in all treatments compared to control (4.173 μm) (*P* < 0. 01) (Fig. 35c).

### Atrium

**Fig. 36:**
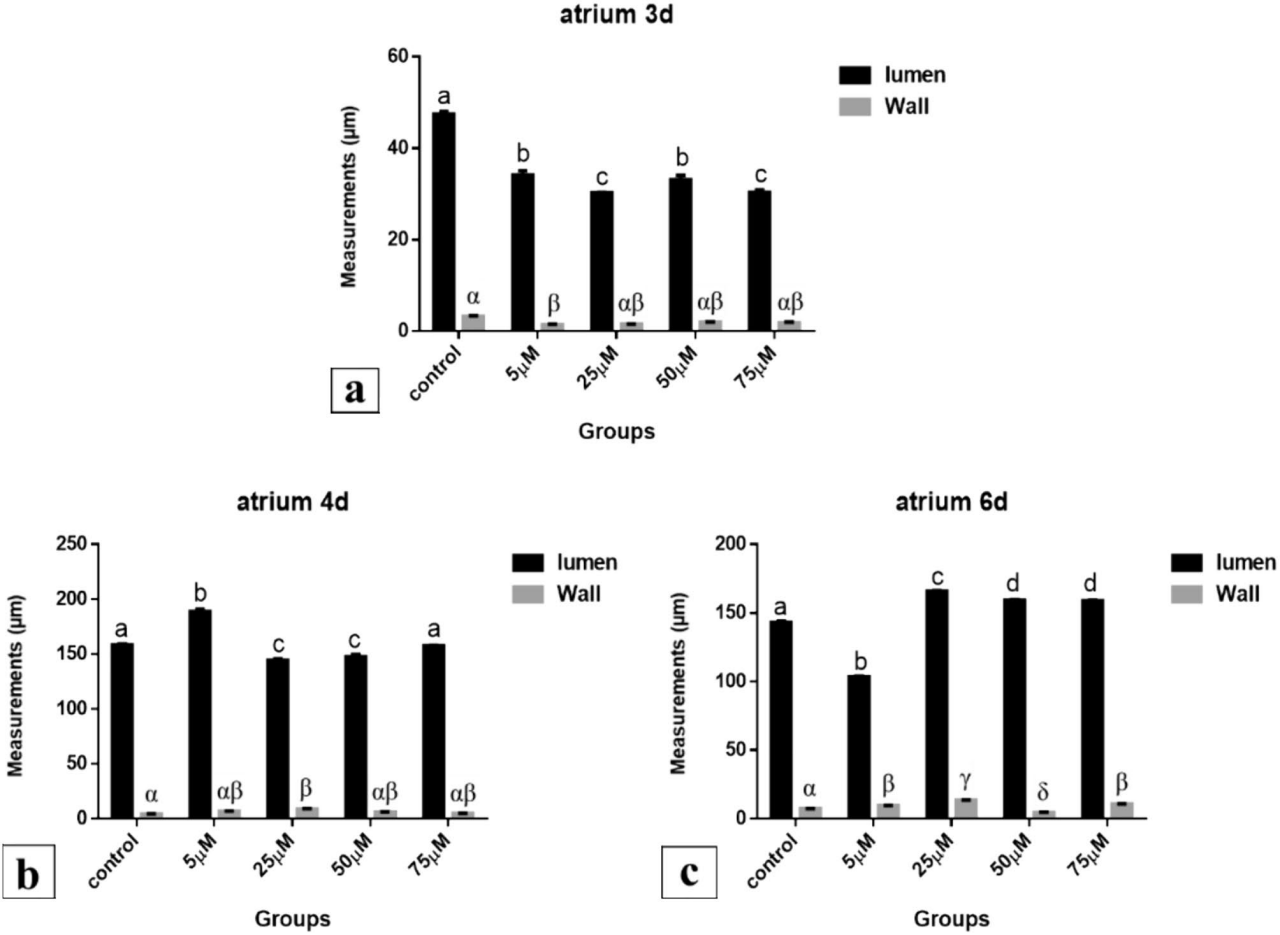
Atrium measurements (µm). A comparison between different treated groups, **a** 72 hours after treatment, **b** 96 hours after treatment, **c** 144 hours after treatment. (a, b, c, d) & (α, β, γ, δ): significant difference comparing to control

*After 72 h of incubation*, morphometric measurements revealed that the lumen of atrium was significantly decreased (34.175, 30.228, 33.118 and 30.388 μm respectively) in all Cd treated groups compared to control (47.450 μm) (*P* < 0.001). The wall of atrium was insignificantly decreased (1.501, 1.918 and 1.849 μm) in 25, 50 and 75 µM Cd treated groups respectively compared to the control (3.237 μm) (*P* < 0.05). The lowest dose (5 µM) of Cd was significantly decreased (1.397 μm) (Fig. 36a).

*After 96 h of incubation*, compared to the control (158.448 μm) the lumen of atrium was significantly increased (188.848 μm) in 5 µM Cd group, while it was significantly decreased (144.552 and 147.573 μm) in 25 and 50 µM Cd groups respectively (*P* < 0.001), and in the 75 µM Cd group the lumen measurement was insignificantly different (157.567 μm), where it was nearly as the normal one (*P* < 0.05). The increase in the lumen of atrium was significant in the lowest dose (5 µM) Cd group compared to the other three doses of the Cd (*P* < 0.001). Atrial wall was insignificantly increased (6.869, 6.016 and 4.744 μm) in 5, 50 and 75 µM Cd treated groups respectively compared to control (4.260 μm) (*P* < 0.05). In the 25 µM Cd treated group, it was significantly increased (8.793 μm) (*P* < 0.05). Among Cd treated groups, morphometric measurements of atrium revealed insignificant difference (*P* < 0.05) (Fig. 36b).

*After 144 h of incubation*, the morphometric measurements of the atrial lumen was significantly decreased (103.374 μm) in 5 µM Cd group compared to control (143.168 μm) and to the other three doses of Cd, while it was significantly increased (165.805, 159.216 and 158.970 μm) in the three higher doses (25, 50 & 75 µM) Cd groups respectively compared to the control (*P* < 0.001). The thickness of atrial wall was significantly increased (9.373, 13.508 and 10.800 μm) in 5, 25 and 75 µM Cd groups respectively (*P* < 0.05, *P* < 0.001 respectively) and significantly decreased (4.490 μm) in 50 µM Cd group compared to control (7.339 μm) (*P* < 0.01) (Fig. 36c).

#### Sinus Venosus

**Fig. 37:**
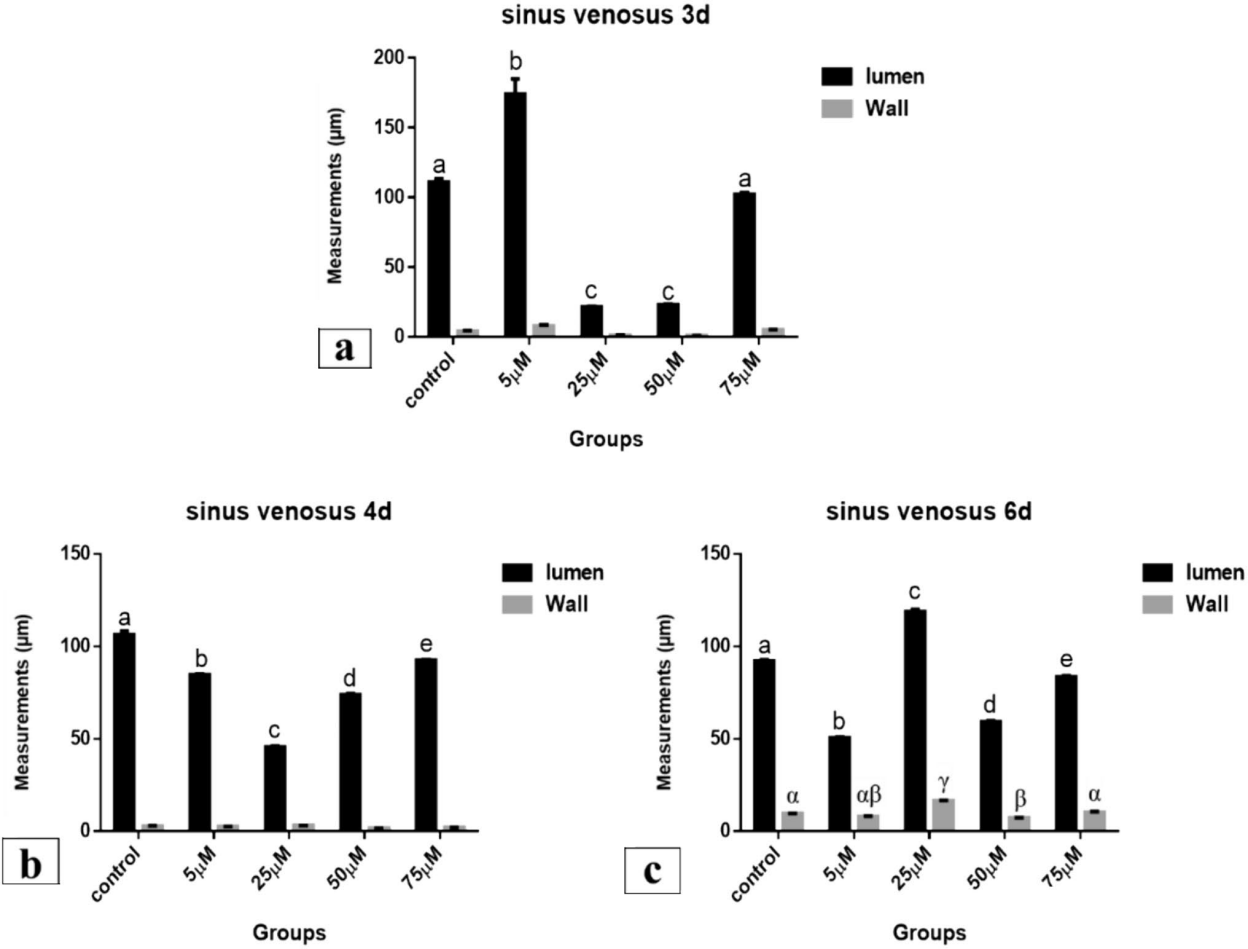
Sinus venosus measurements (µm). A comparison between different treated groups, **a** 72 hours after treatment, **b** 96 hours after treatment, **c** 144 hours after treatment.(a, b, c, d, e) & (α, β, γ): significant difference comparing to control

*After 72 h of incubation*, morphometric measurements of the sinus venosus lumen revealed that there was highly significant increase (173.851 μm) in 5 µM Cd group with compared to control (111.087 μm) and other Cd doses (*P* < 0.001). In the other hand, there was highly significant decrease (21.520 and 23.165 μm) in 25 and 50 µM groups respectively (*P* < 0.001) but in 75 µM group there was insignificant decrease (102.211 μm) compared to control (*P* < 0.05). The morphometric measurements of the sinus venosus wall were insignificantly increased (7.863 and 5.033 μm) in 5 and 75 µM Cd groups respectively and insignificantly decreased (1.231 and 1.044 μm) in 25 and 50 µM groups respectively compared to control (4.267 μm) (*P* < 0.05) (Fig. 37a).

*After 96 h of incubation*, the lumen of sinus venosus was highly significant decreased (84.734, 45.650, 73.961 and 92.532 μm respectively) in all treatments compared to the control (106.286 μm) (*P* < 0.001). Also, the decrease between the treated groups was significant (*P* < 0.001). The wall of sinus venosus showed insignificant decrease (2.531, 1.634 and 2.134 μm) in 5, 50 and 75 µM Cd groups respectively. In 25 µM Cd group, it was insignificantly increased (3.057 μm) compared to control (2.838 μm) and to each other (*P* < 0.05) (Fig. 37b).

*After 144 h of incubation*, the lumen of sinus venosus was highly significant decreased (50.556, 59.342 and 83.591 μm) in 5, 50 and 75 µM Cd groups respectively. 25 µM Cd group, revealed a significant increase (118.869 μm) compared to control (92.166 μm) and to other treated groups (*P* < 0.001). In addition, the decrease in the lumen was significant among treated groups (*P* < 0.001). The wall of sinus venosus was highly significantly increased (16.604 μm) in thickness in 25 µM group and significantly decreased (7.301 μm) in 50 µM group compared to control (9.635 μm) (*P* < 0.001, *P* < 0.05 respectively). The 5 and 75 µM groups revealed no significant difference (8.062 and 10.329 μm respectively) in the wall of sinus venosus compared to control (*P* < 0.05) (Fig. 37c).

### Dorsal Aorta

**Fig. 38:**
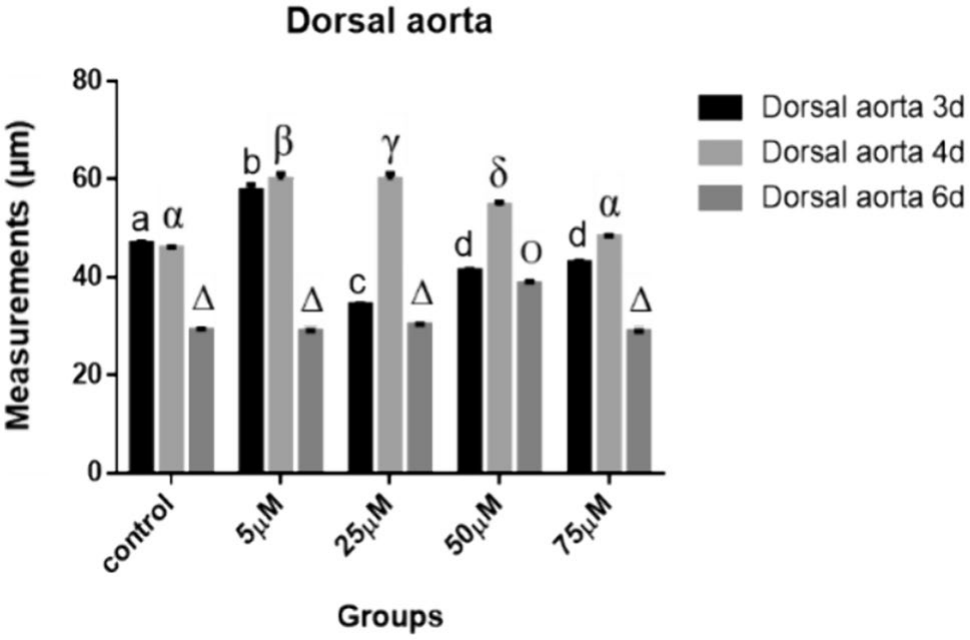
Dorsal aorta measurements (µm). A comparison between different treated groups, 72 hours, 96 hours and 144 hours after treatment. (a, b, c, d), (α, β, γ, δ) & (Δ, О): significant difference comparing to control

*After 72 h of incubation*, the morphometric measurements of dorsal aorta lumen significantly decreased (34.358, 41.214 and 42.971 μm) in 25, 50 and 75 µM groups respectively. 5 µM group revealed a significant increase (57.564 μm) compared to control (46.932 μm) (*P* < 0.001) (Fig. 38).

*After 96 h of incubation*, 5, 25 and 50 µM groups revealed a significant increase (59.884, 59.884 and 54.537 μm respectively) in the lumen of dorsal aorta (*P* < 0.001), while insignificant increase (48.176 μm) in 75 µM group compared to control (45.997 μm) (*P* < 0.05) (Fig. 38).

*After 144 h of incubation*, the morphometric measurements of the dorsal aorta lumen revealed no significant difference (28.886, 30.226 and 28.847 μm) in 5, 25 and 75 µM Cd groups respectively. 50 µM group showed a significant increase (38.616 μm) in the dorsal aorta lumen compared to control (29.075 μm) and to other treatments (*P* < 0.05, *P* < 0.001 respectively) (Fig. 38).

#### Heart and Respiratory Rate Measurements

**Fig. 39:**
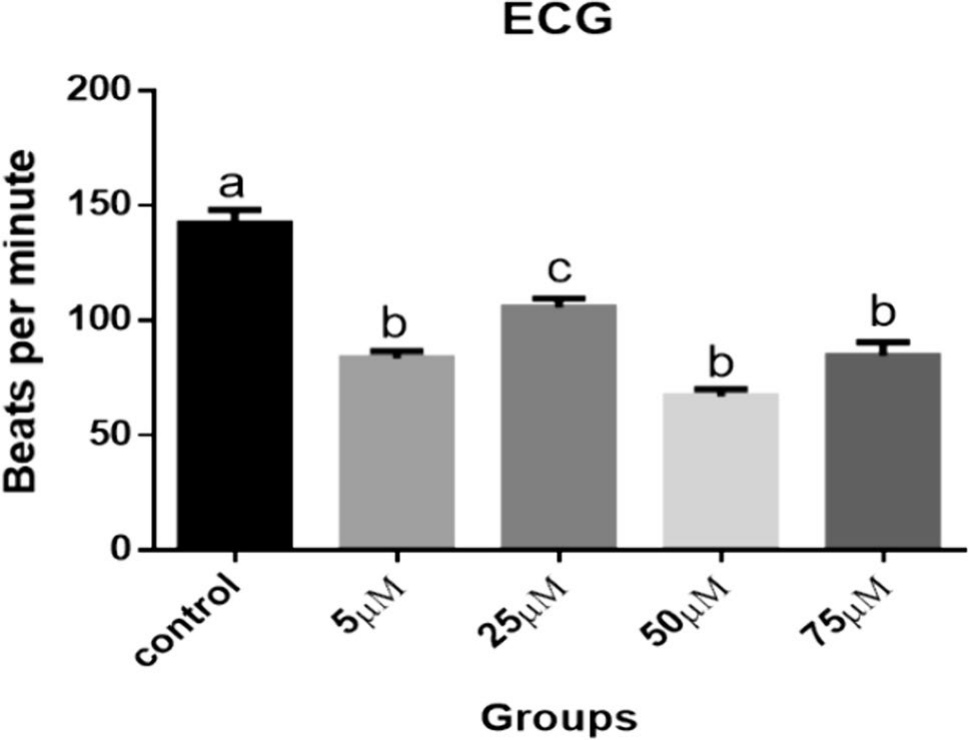
Heart rate measurements (beats per minute). A comparison between different treated groups, ten days after treatment. (a, b, c): significant difference comparing to control

**Fig. 40:**
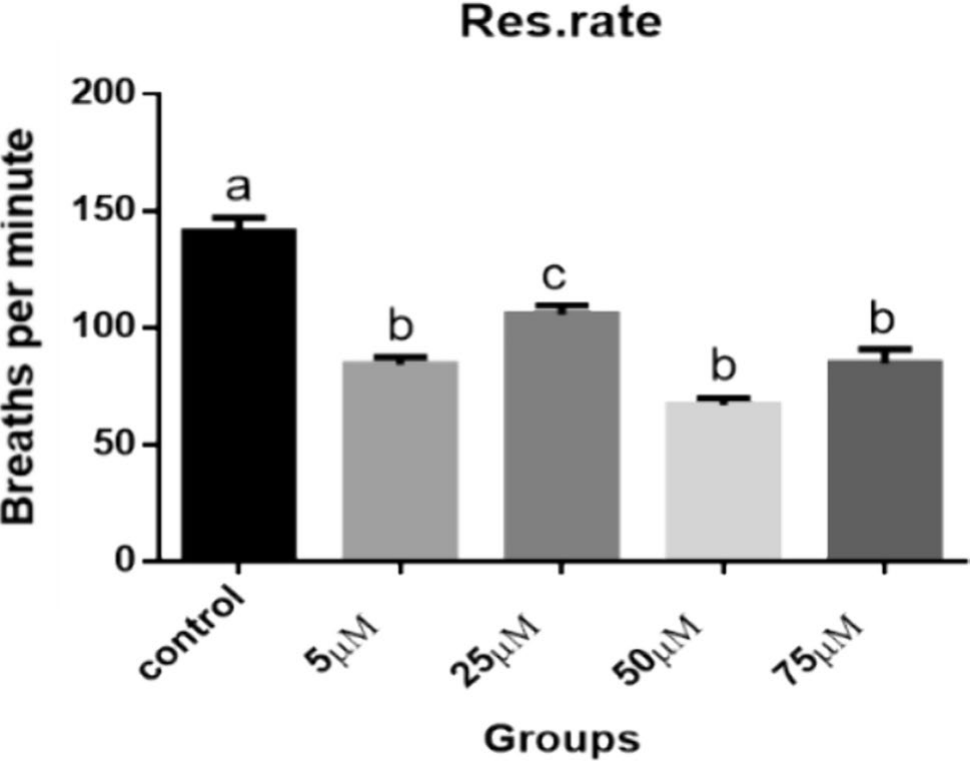
Respiratory rate measurements (breaths per minute). A comparison between different treated groups, ten days after treatment. (a, b, c): significant difference comparing to control

Figures 39 and 40 indicate that, at the embryonic age of ten days, all the Cd treated groups showed a significant decrease in the pulse of the heart (83.50, 105.8, 67.03, 84.70 beats/minute respectively) compared to control (142.2 beats/minute) and consumption of oxygen (84.17, 105.8, 67.03 and 84.70 breaths/minute respectively) compared to control (141.1 breaths/minute) (*P* < 0.001), while among Cd treated groups there was insignificant difference (*P* < 0.05) except in 25 µM group which showed significant increase (*P* < 0.01).

## Discussion

The present study revealed that Cd treatment induced structural damage in the heart chambers and dorsal aorta and reduced heart work and oxygen consumption in the developing chick embryos. The development of the cardiovascular system begins early during embryonic growth, and exposure to several environmental pollutants can cause abnormalities in the development and function of the cardiovascular system [30]. Also, many previous studies reported that such pollutants can induce inflammation, which can result in cardiovascular damage [31]. Cd is one of cardiotoxic material which destroys myocardial cells directly and release biomarkers of cardiac tissue damage into the blood system like LDH [32], implying certain cardiac damage such as myocarditis.

There is lots of information linking cardiovascular disease risk to Cd exposure [33–35]. However, the link between congenital heart defects and Cd exposure is under-studied. The present study showed that different concentrations of Cd has a negative effect on the cardiac muscle. In younger ages, Cd-induced small size of cardiac muscle and thickening of the epimyocardium. Upon serial examination of the cardiac muscle during the embryonic development of the chick treated with Cd, it was found that it causes enlargement of the heart and thinning of epimyocardium. Necrosis and inflammatory cells appeared between the muscle fiber, in addition to edema, myocardial infraction, congestion of blood vessels and also degradation of muscle fibers. Also, the morphometric measurements of heart chambers during chick development reflected Cd toxicity. Heart rate and respiratory rate were reduced as a result of heart damage. This finding is consistent with other studies that reported Cd-induced hypertrophy of cardiac muscle through thickening of the ventricles of the heart [36, 37]. As stated by Wang et al. [38], Zhang et al. [39] and McCauley et al. [37], this thickening may be due to increased cell proliferation associated with higher expression levels of the essential cell cycle genes. Furthermore, in rats exposed to Cd, heart hypertrophic remodeling is related to these alterations in embryonic gene expression [36]. On the other hand, Lin et al. [40] demonstrated that Cd exposure caused cardiac morphological damage characterized by myocardial hemolysis, widening of myocardial space, rupture of myocardial fibers and up-regulation of pro-apoptotic genes indicating that Cd exposure initiated myocardial apoptosis. According to a study by Pawlak et al. [41], Cd has adverse effects on the morphometric components of chick embryos’ hearts. Measurements showed significant decrease in heart weight and thickness of the ventricle wall, in embryos exposed to Cd [42]. This result is in parallel with the present finding in which Cd toxicity induced apoptotic cells making cardiac muscle smaller in size and the epimyocardium wall thinner in thickness. The effect of Cd on heart weight looked to be dose dependent. The present study revealed that Cd induced malformations and splitting in the dorsal aorta. This may be due to the alteration in Angiopoetin-2 (Ang-2) and VE cadherin, a trans-membrane protein associated with adherens-type intercellular junctions, which caused delay in chick embryo vasculogenesis and impaired angiogenesis as reported by Gheorghescu & Thompson [27]. Cheng et al. [43] used the 3D reconstruction of microangiographs to illustrate the impact of Cd exposure on developmental defects of the vasculature of zebra fish embryos. They observed defective vascular patterning and small vessels. The dorsal and ventral aortae running along the length of the trunk region broke up near the tails, while collateral vessels were developed in the surroundings. Their observation of development of collateral vessels might explain the observation of splitting of dorsal aorta in the current study. They suggested that Cd may interfere with other essential divalent ions such as Mg^+2^ which is essential for activation of transcription factors required for angiogenesis. Additionally, Oliveira et al. [44] found that Cd exposure decreased the vasodilatation response of aortic ring of mice, suggesting reduction in nitric oxide (NO) bioavailability and inducing endothelial dysfunction. In that same context, we report here that Cd treatment induced significant changes in the availability of NO during chick embryo development (unpublished data). Furthermore, Angeli et al. [45] stated that by Cd induced dysfunction, the aorta is a main site to be affected by oxidative stress. They found that Cd decreased vasorelaxation of mice aortic rings through endothelial injury. While, Gökalp et al. [46] found a significant inhibition of relaxation response to Acetylcholine in aortic rings of Cd-hypertensive treated rats. They suggested that the reduction in endothelium-dependent relaxation might be due Cd-induced hypertension. Else, Zhang et al. [47] stated that Cd resulted in ferroptosis (a novel form of cell death that is triggered by lipid peroxidation) of vascular endothelial cells in vivo and in vitro. In the visualized zebrafish embryos, Cd causes the death of vascular endothelial cells through necrosis, apoptosis and autophagy. The toxicity of Cd to cells is closely related to oxidative stress. Cd exposure increases the excessive accumulation of ROS through consumption of glutathione (GSH), inactivation of NADPH oxidase and reduction of the activity of antioxidant enzymes such as superoxide dismutase and catalase, leading to cell death. They suggested that Cd induced ferroptosis through upregulation of Heat Shock Protein 70 (Hsp70), which might affect several targets leading to endothelial injury. It is suggested here that the Cd induced disturbances in dorsal aorta development might be explained as a result of affecting factors required for vasculogenesis, alterations in the elements essential for vasorelaxation or vasoconstriction, inducing oxidative stress and consequently enhancing endothelial injury or accelerating cell death via activation of ferroptosis. The present work is coinciding and confirm the conclusion of Cheng et al. [43], Oliveira et al. [44], Angeli et al. [45], Gökalp et al. [46] and Zhang et al. [47].

The present results revealed that the effect of Cd on lumen width of heart compartments was dose dependent. The increase in Cd concentration, the wider truncus arteriosus, ventricle, atrium, sinus venosus or dorsal aorta. This dose depended effect was clearly obvious in the early stages rather than late stages. It is suggested here that the widening of heart compartments might be due to the damage in the cardiomyocytes of heart compartments walls which might be resulted in less firm and loose muscular structures. In their study, Roy et al. [48] found that Cd negatively affected the viability of endothelial cells and embryonic fibroblasts and induced cell death in a dose dependent manner. Li et al. [49] found that Cd accumulated in the developing Japanese quill heart in a dose dependent manner. The accumulated Cd caused microstructural and ultrastructural cardiac damages resulting in myocardial dysfunction. Ali et al. [50] reported that hematological and biochemical parameters and body weight significantly decreased in a dose-dependent manner in the broiler birds. They concluded that alterations in hematological and biochemical markers might significantly contribute to systemic toxicity in broilers. The present work is coinciding and support the conclusion of Roy et al. [48], Li et al. [49] and Ali et al. [50] and confirm the dose dependent cardiotoxic effect of Cd on the developing chick heart.

The current study indicated that low Cd doses impaired heart development in the early stages of embryonic ages rather that the higher doses. This may be explained as the low Cd dose was under the threshold concentration required to trigger embryonic defense mechanism or it was matching the concentration required to initiate the apoptotic genes, while higher doses were enough to trigger the immune defense system and hence, the higher doses had less prominent effect in inducing heart abnormalities. To our knowledge, there is a shortage of information concerning the differential effects of different Cd concentration on heart congenital anomalies. Further research is recommended in this aspect. Also, the current study revealed that at the embryonic age of 144 h the heart had more or less normal architecture in all groups, despite noticeable deviations in younger ages. Thompson et al. [51] found that chick embryos treated with 50 µM Cd acetate experienced a significant down-regulation of SOD and other antioxidants one hour after Cd treatment. However, later on, the expression levels of SOD and other antioxidants had recovered and even surpassed those of control embryos. The expression levels of all antioxidants had returned to normal. This means that the immune defense system had relived and had been able to diminish the Cd toxicity effect on at least heart architecture regardless of its effect on either heart weight or heart efficiency. It is suggested here that Cd embryo toxicity is more prominent in early stages of development rather than later stages due to the recovery of the immune defense mechanism in older ages and diminishing Cd toxicity.

Examination of the heart muscle of the recently hatched chicks revealed that all of the Cd treated groups had various abnormalities. These results match with those of previous research [41, 52, 53]. The histological anomalies in the heart muscle have numerous causes. According to Weiss et al. [54] and Li et al. [55] there is a close relationship between cardiac damage and inflammation. The fluctuation of intracellular inflammatory cytokines level was deemed as a cardiac injury marker, which has been increasingly recognized. So, inflammatory intracellular infiltration was found in cardiomyocytes that were exposed to Cd. Additionally, Yazihan et al. [42] reported that, in rat hearts, exposure to Cd causes apoptosis and inflammation, which was assessed using a variety of specialized biomarkers. Increased apoptosis was noted in the cardiac tissues of the Cd exposed embryos, which is consistent with previous findings [15, 56–58]. Myocardial fibrosis may be induced by cardiomyocyte death [59].

Also, damage of heart tissue induced by Cd may be due to triggering oxidative stress. Oxidative stress has been suggested as one of the major mechanisms of Cd-induced multi-organ toxicity by de Boer et al. [60] and Meng et al. [61]. According to Yu et al. [62] oxidative stress caused by Cd resulted in damage to cardiac tissue in chicken heart. The effect of Cd on development may be related to the production of free radicals and the deficiency of antioxidant defense system [63]. The heart in particular is very sensitive to oxidative stress [64]. Cardiac oxidative stress can intensify heart failure and promote disease progression [65].

The present data showed that heart work and respiratory rate were negatively affected due to Cd exposure. The effect of Cd toxicity on heart beats and respiratory rate seems to be dose dependent, where the higher two doses resulted in more reduction in heart efficiency. Heart rate of avian embryos is one of the most often reported parameters of cardiac work [66, 67]. A significant decrease in the heart rate of the chicken embryos exposed to different concentrations of CdCl_2_ during development was explained in the final stage of incubation probably due to the embryo absorbing and accumulating Cd injected into ovalbumin [41, 68]. The same result was confirmed in vitro [69]. Liu et al. [70] noticed that the heart rate of the 72 hpf (hours post fertilization) zebrafish larvae exposed to CdCl_2_ was significantly reduced by increasing cellular apoptosis and production of inflammatory factors.

Another possible mechanism is connected with calcium, one of the elements responsible for normal heart work [71]. It is known that Cd may interfere with calcium metabolism by removing it from the body [72]. In addition, Cd is a blocker of calcium channels [73, 74]. Cd influences the renin-angiotensin system’s activity through a number of metabolic pathways [75]. As a result, Cd may lower the concentration of angiotensin II, which is created when the dipeptide carboxylase acts on angiotensin. This compound’s lower concentration inhibits the AT-1 receptor from being stimulated, which increases the amount of calcium that enters heart muscle cells and releases calcium from the sarcoplasmic reticulum [75]. It is suggested here that the reduced heart work and respiratory rate of Cd exposed embryos after ten days of incubation might be due to induction of apoptosis and inflammation in cardiac muscle, triggering oxidative stress or interfering with calcium metabolism resulting in heart tissue damage and decreasing heart efficiency.

Chick embryo development is a closed independent system away from maternal interference. So the present results might differ in mammals or human. Further studies are recommended to clarify the effect of Cd on human heart work and respiratory rate.

## Conclusion

Overall, the findings of this study suggested that exposure of chick embryos to different low doses of Cd affects the crucial stages of myocardium development, which could predispose the offspring to cardiovascular diseases in the future. This study indicated that Cd exposure induced embryonic cardiomyocyte inflammation and thickening and hyperplasia of cardiac muscles. In addition to this Cd, causes cardiac underdevelopment as well as microstructural and histopathological damages which was reflected on a reduced heart efficiency. The cardiotoxic effect of Cd on heart compartments structure and function is dose dependent.

## Data Availability

No datasets were generated or analysed during the current study.
